# Microbiota in health and diseases

**DOI:** 10.1038/s41392-022-00974-4

**Published:** 2022-04-23

**Authors:** Kaijian Hou, Zhuo-Xun Wu, Xuan-Yu Chen, Jing-Quan Wang, Dongya Zhang, Chuanxing Xiao, Dan Zhu, Jagadish B. Koya, Liuya Wei, Jilin Li, Zhe-Sheng Chen

**Affiliations:** 1 Department of Endocrine and Metabolic Diseases, Longhu Hospital, The First Affiliated Hospital of Medical College of Shantou University, Shantou, Guangdong 515000 China; 2 Department of Pharmaceutical Sciences, Institute for Biotechnology, College of Pharmacy and Health Sciences, St. John’s University, Queens, NY 11439 USA; 3 Microbiome Research Center, Moon (Guangzhou) Biotech Ltd, Guangzhou, 510535 China; 4 School of Pharmacy, Weifang Medical University, Weifang, Shandong 261053 China; 5 Department of Cardiovascular, The Second Affiliated Hospital of Medical College of Shantou University, Shantou, Guangdong 515000 China

**Keywords:** Microbiology, Endocrine system and metabolic diseases

## Abstract

The role of microbiota in health and diseases is being highlighted by numerous studies since its discovery. Depending on the localized regions, microbiota can be classified into gut, oral, respiratory, and skin microbiota. The microbial communities are in symbiosis with the host, contributing to homeostasis and regulating immune function. However, microbiota dysbiosis can lead to dysregulation of bodily functions and diseases including cardiovascular diseases (CVDs), cancers, respiratory diseases, etc. In this review, we discuss the current knowledge of how microbiota links to host health or pathogenesis. We first summarize the research of microbiota in healthy conditions, including the gut-brain axis, colonization resistance and immune modulation. Then, we highlight the pathogenesis of microbiota dysbiosis in disease development and progression, primarily associated with dysregulation of community composition, modulation of host immune response, and induction of chronic inflammation. Finally, we introduce the clinical approaches that utilize microbiota for disease treatment, such as microbiota modulation and fecal microbial transplantation.

## Introduction

The origin of “microbiota” can be dated back to early 1900s. It was found that a vast number of microorganisms, including bacteria, yeasts, and viruses, coexist in various sites of the human body (gut, skin, lung, oral cavity).^1^ In addition, the human microbiota, also known as “the hidden organ,” contribute over 150 times more genetic information than that of the entire human genome.^2^ Although “microbiota” and “microbiome” are often interchangeable, there are certain differences between the two terms. Microbiota describes the living microorganisms found in a defined environment, such as oral and gut microbiota. Microbiome refers to the collection of genomes from all the microorganisms in the environment, which includes not only the community of the microorganisms, but also the microbial structural elements, metabolites, and the environmental conditions.^3^ In this regard, microbiome encompasses a broader spectrum than that of microbiota. In the current review, we mainly focus on the function of microbiota in human health and diseases.

**Fig. 1:**
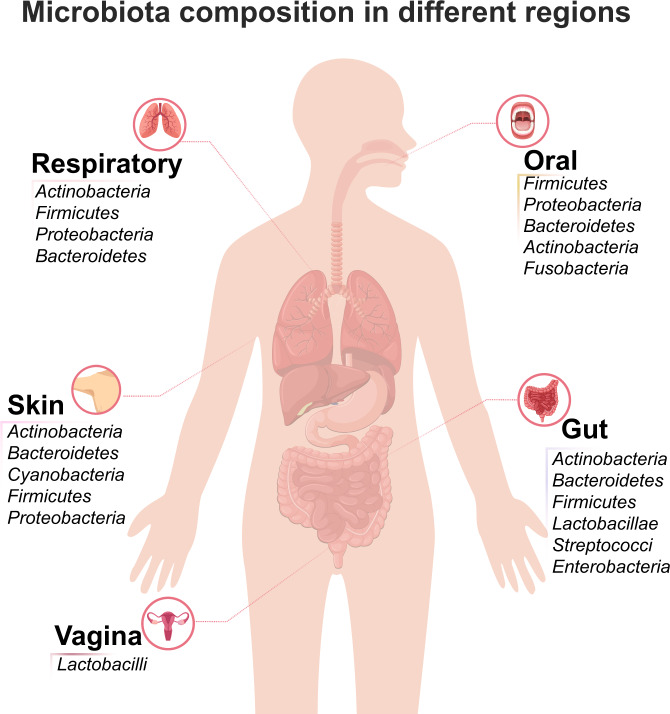
Human microbiota composition in different locations. Predominant bacterial genera in the oral cavity, respiratory tract, skin, gut, and vagina are highlighted

The composition of microbiota varies from site to site (depicted in Fig. 1). Gut microbiota is considered the most significant one in maintaining our health.^4^ The gut bacteria serve several functions, such as fermentation of food, protection against pathogens, stimulating immune response, and vitamin production.^5^ Generally, the gut microbiota is composed of 6 phyla including *Firmicutes*, *Bacteroidetes*, *Actinobacteria*, *Proteobacteria*, *Fusobacteria*, and *Verrucomicrobia*, among which *Firmicutes* and *Bacteroidetes* are the major types.^6^ The most studied fungi (gut mycobiota) are *Candida, Saccharomyces, Malassezia, and Cladosporium.*^7^ In addition to bacteria and fungi, the human gut microbiota also contain viruses, phages, and archaea, mainly *M. smithii.*^8^

While less well established compared with gut, microbiota is also localized in other regions including the oral cavity, lung, vagina, and skin. Oral microbiota is considered the second largest microbial community in human.^9^ The oral cavity can be further divided into multiple habitats of microbiota, including saliva, tongue, tooth surfaces, gums, buccal mucosa, palate, and subgingival/supragingival plaque, which may exhibit substantial and rapid changes in composition and activity, owing to the factors such as changes in pH, gene mutations, and interactions among the bacteria.^10^ The microbiota composition in all seven sites shares overall similarities but with small scale differences. In general, the major bacteria present in oral microbiota are *Firmicutes*, *Proteobacteria*, *Bacteroidetes*, *Actinobacteria*, and *Fusobacteria*.

Although healthy human lungs were long considered sterile, numerous studies have demonstrated that microbiota is also present in lung tissues.^11^ The core lung microbiota included *Actinobacteria*, *Bacteroidetes*, *Firmicutes*, and *Proteobacteria*. The composition of lung microbiota is primarily determined by three factors: 1) microbial immigration, 2) the elimination of microorganisms, and 3) the reproduction rates of microorganisms.^12^

In human skin, the distribution and variety of glands and hair follicles vary among each geographic region. The physical and chemical differences of skin regions create distinct composition of microbiota.^13^ Generally, the skin microbiota is composed of *Actinobacteria*, *Bacteroidetes*, *Cyanobacteria*, *Firmicutes*, and *Proteobacteria*.

In recent decades, tremendous amount of work has highlighted the relationship between microbiota and diseases such as cancers, diabetes, and neurological disorders. Moreover, manipulating microbiota in human body can be key for disease treatment. Here, we summarize and discuss the current state of knowledge of human microbiota in development of diseases, mediating health conditions, and the potential clinical application in disease treatments.

## Microbiota in health

### The “healthy” gut microbiota

Intestinal microbial balance is closely relevant to human diseases and health. Compared with other regions of the body, the human gastrointestinal (GI) tract contains an abundant microbial community which gathers ~100 trillion microorganisms.^14^ Extensive studies have been performed to reveal the important relationship between gut microbiota and basic human biological processes. For example, current advances have shown that human microbiota is closely involved in nutrient extraction, metabolism, and immunity.^15^ Microbiota may affect biological processes via several mechanisms. For energy and nutrient extraction from food, microbiota plays crucial roles due to the versatile metabolic genes which provide independent unique enzymes and biochemical pathways.^16^ Moreover, the biosynthesis of bioactive molecules such as vitamins, amino acids and lipids, are also highly dependent on the gut microbiota.^17^ Regarding the immune system, the human microbiota not only protects the host from external pathogens by producing antimicrobial substances but also serves as a significant component in the development of intestinal mucosa and immune system.

In healthy conditions, the gut microbiota exhibits stability, resilience, and symbiotic interaction with the host. There is a lot of research into the definition of a “healthy” gut microbiota and its link to host physiological functions. Gut microbiota is composed of bacteria, yeasts, and viruses. A healthy microbiota community often demonstrates high taxonomic diversity, high microbial gene richness and stable core microbiota.^18^ However, it should be noted that the relative distribution of microorganisms is unique between individuals and may undergo variations within the same individual. In human, gut microbiota may vary due to age and environmental factors (for example, medication usage). Additionally, gut microbiota varies in different anatomical parts of the GI tract. For example, *Proteobacteria* such as *Enterobacteriaceae* are found in the small intestine but not the colon. Instead, *Bacteriodetes* such as *Bacteroidaceae*, *Prevotellaceae* and *Rikenellaceae* are often found in the colon.^19^ Such variations are majorly due to the different environments. In the small intestine, the transit time is short and bile concentration is high, while in the colon, which has slower flow rates and milder pH, as well as larger microbial communities, especially anaerobic types, are commonly observed.^20^ Besides spatial distribution, gut microbiota also differs by age. Generally, the microbiota diversity increases in the time between childhood and adulthood and decreases at older age (over 70).^21^ Before the formation of a relatively stable gut microbiota composition, the diversity of children’s microbiota is dominated by *Akkermansia muciniphila*, *Bacteroides*, *Veillonella*, *Clostridium coccoides* spp., and *Clostridium botulinum* spp.^22^ At about age 3, children’s gut microbiota becomes comparable to that of adults, with three major microbial phyla including *Firmicutes*, *Bacteroidetes* and *Actinobacteria* becoming dominant.^23^ Subsequently at older age, dietary and immune system change potentially affect the composition of the human gut microbiota. Specifically, elder people tend to exhibit decreased *Bifidobacterium* and increased *Clostridium* and *Proteobacteria.*^24^ The decrease in the anaerobic bacteria *Bifidobacterium* is considered relevant to deteriorated inflammatory status due to its role in stimulating the immune system. Since the microbiota plays an important role in human well-being, also proactively involves in multiple biological processes and disease development, the research on human microbiota is going beyond compositional studies and investigation on members’ associations. Specifically, more attention has been paid on explaining the causality of microbiota functions, especially with the boom of new techniques of high-throughput sequencing, microbiota interactive modeling and simulation. Overall, further investigations are still necessary to unveil the roles of human microbiota, in order to support the development of microbiome-based diagnosis and personalized medicine (Table 1).

**Table 1:** Mouse models in microbiota research

Mouse models	Research field	Significance
Germ-free mice colonized with human microbiota^410,411^	host-microbiota relationship in different systems, including GI tract, cardiology, reproductive biology, lipid metabolism, and bone homeostasis.	Free of all microorganisms and allow colonization with specific microbiota.
		Normal host physiologic parameters are altered
Antibiotic treated^412^	Antibiotics can be used to deplete specific member of microbiota, allows for the study of the role of bacteria in maintaining cell functionality and signaling pathways after development.	Applicable to any genotype or condition of mouse.
		May cause selection of drug-resistant bacteria.
Genetically modified^413,414^	Resemble the phenotype associated with genetic defects in diseases such as IBD.	Provides a powerful tool to study the pathogenic mechanisms of human diseases.
		Genes that involve in multiple pathways may interfere the result.
Chemical modified^415^	Using chemicals to damage gut epithelial cells, or to induce immune response in the mucosa.	A common way to induce colitis in mice.
		May result in contradicted result with variation in experimental design and environmental factors.

### Rodent models for human microbiota research

The human microbiota has attracted more and more research in recent decades. However, the studies of local microbiota require invasive sampling methods, with practical or ethical reasons in concern. Animal models, particularly mouse and rat models, have also been used to study the pathogenic and therapeutic potential of microbiota with varies diseases.^25^ With a majority of microbiota research is focusing on gut microbiota, the use of germ-free (GF) mouse model has become popular due to its translatability. It should be noted that, in order to translate such generated knowledge from rodent to human, the similarities and differences between their microbiota profile need to be considered. In Table 2, we summarized some commonly used rodent models and their role in microbiota research.

**Table 2:** Summary of pathogenic microbiota and the related signaling pathways

Diseases	Significant pathogens	Related signaling pathways
Cardiovascular diseases	*T. forsythia, P. gingivalis*	Inflammatory mediators IL-6, CRP, LPS, SCFAs MAPK and NF-κB signaling pathways
Cancer	*P. gingivalis, F. nucleatum*, *T. forsythia, E. faecalis*, *E. coli*, *B. fragilis*	Induced chronic inflammation and oncometabolites production. NF-κB, JAK1/STAT3, PI3K, Wnt/β-catenin signaling pathway
Diabetes mellitus	*R. faecis*, *F. prausnitzzi*, *C. coccoides, E. rectale*	Increased proinflammatory cytokines IL-1β, IL-6, and TNF-α Decreased anti-inflammatory cytokines IL-10 and IL-13 TLR4/MyD88 pathway NF-κB, IL-6 and TNF-α pathways
Chronic respiratory diseases	*S. pneumonia*, *H. influenza*, *M. catarrhalis F. prausnitzii*, *R. mucilaginosa, M. salivarium*	Th17/IL-17-mediated inflammation TNF-α, IL-6, IFN-γ, and IL-17A pathways
Inflammatory bowel diseases	*E. coli, H. pylori*	IL-6, TNF-α, CXCL2
Chronic kidney diseases	*P. gingivalis*, *T. denticola*, *A. actinomycetemcomitans*, *T. forsythia, T. denticola, E. coli*	TMAO
Chronic liver diseases	*Gammaproteobacteria, Erysipelotrichi, P. gingivalis*	TGF-β signaling pathway TLR4/MyD88-NF-κB-dependent pathway

The genome data showed that more than 85% of the genomic sequences between human and mouse are conserved, while the main difference is found in the primary sequence of regulatory elements. Cheng et al. reported that, in murine genome, half of the transcription factor binding sites may not have orthologous sequences in human genome.^26^ Moreover, the genomic studies have shown a significant difference in the immune system and its regulation in different species. Since gut microbiota has major impact to host innate and adaptive immune responses, the translation of findings from rodents to human should be carefully validated before drawing definite conclusions. While human and murine gut microbiota has 90% overlapping in phyla and genera levels, the composition and abundance of microbes have key discrepancies.^27^ For instance, the major difference is the Firmicutes/Bacteroidetes ratio, where it is significantly higher in human than mice. Particularly, human Bacteroidetes is mainly composed of *Prevotellaceae* and *Bacteroidaceae*, while mice Bacteroidetes are primarily composed of *S24-7*. Regarding the Firmicutes, *Ruminococcaceae* is the major phylum observed in human and *Clostridiales* is the major one observed in mice. Moreover, human and mouse each carries specific genera, such as *Faecalibacterium, Megasphera, Asteroleplasma, Succinivibrio, Paraprevotella* in human and *Mucispirillum* in mouse.^28^

### Colonization resistance

Humans are born with and form a large community of symbiotic and pathogenic microbes, which inhabit our gut, skin, mucosal passages, and form a stable community that is resistant to external pathogens. The term “colonization resistance” was initially coined in the 1950s when Bohnhoff et al. found that mice became significantly sensitive to a specific type of bacterial infection after antibiotic treatment.^29^ Later, such conclusion was further applied to the phenomenon that current microbiota could provide resistance the colonization of invading pathogenic species, also from which researchers recognize. As a result, the microbiota is crucial shield in protecting us from exogenous microorganisms. Despite the fact that microbiota colonization resistance has not been fully elucidated, with the advent of GF animal models, researchers have discovered several potential mechanisms such as nutrient competition,^30^ antimicrobial production, and bacteriophage deployment. Another example of colonization resistance is the interaction of symbolic and pathogenic *E. coli*., where indigenous *E. coli* strains compete with pathogenic *E. coli* O157:H7 for the amino acid proline in consuming nutrients.In this section, we focus on the gut microbiota and colonization resistance. The vaginal and skin microbiota and their colonization resistance are also discussed.

The GI tract digests proteins as well as sugars from foods. Metabolizing polysaccharides and specific proteins requires multiple enzymes produced by various bacteria. For example, *Bacteroides* species in the large intestine are responsible for sugar harvest.^31^ Pathogenic *Enterobacteriaceae* also utilizes sugar and amino acids in gut.^32^ Freter et al. proposed a niche hypothesis which has been supported by in vitro and in vivo studies. The hypothesis states that the composition and abundance of gut microbiota is determined by one or a few nutritional substrates.^33^ In mouse models, when a single type of sugar is removed, both the microbiota composition and the ability of resistance to pathobiont were altered.^34^

Probably due to the necessity of competing with foreign bacteria, gut bacteria have developed various ways of suppressing competitors, including the secretion of diverse bacteriocins. A contact-dependent competition in the gut, namely type 6 secretion system, was originally identified in the bacteria secretion system involved with eukaryotic cells,^35^ which was later found relevant to intraspecies killing. The system works by contact cells delivering effectors, such as degraders of nucleotide, cell walls and membranes, into the cytoplasm.^36^ Moreover, this system may also contribute to the abundance of *Bacteroides* species in the mouse and human gut.^37^ Besides the type 6 system, other systems such as type 7 (or ESX system), also mediate the intra- and interspecies killing.^38^ Currently, the contact-dependent systems of gut microbiota inhibition and growth are being increasingly discovered. The intermediate genes, immunity and effectors may serve as amenable factors which are modifiable via bioengineering methods. Additionally, they are valuable for studying the interactions, structure, and dynamics of the gut microbiota. However, the exact role of bactericidal mechanisms remain poorly understood and further studies are still necessary.

Bacteriophage deployment is another mechanism of colonization resistance in the gut; however relevant research is still in an immature stage.^32^ It has been revealed that two cycles, namely the lytic cycle and the lysogenic cycle, are involved in bacteriophage infection. Phages duplicate by injecting genomic segment into the bacterial cytoplasm, after which the two cycles start to branch. Phages in lysogenic stage insert their genome into bacteria genome and render prophages, which guarantees the replication of phage DNA and entrance into the lytic cycle. In the lytic cycle, phage DNA starts replication, modification and expression, resulting in new phage assembly, cell lysis and phage spreading.^39^ There are several potential mechanisms to prevent bacteriophage infection including the blockage of surface receptor recognition, superinfection exclusion system and abortive infection. For infection prevention, resistant strains exhibit compositions similar to bacterial surface and thereby could serve as decoys for attacking phages.^40^ DNA replication could be prevented by “restriction-modification” system, mainly by methyltransferase and restriction endonucleases. This system serves as the primitive inner bacteria defense system in the human body, despite the disadvantage of this system that it also damages the host DNA.^41^ The defense system of bacteria also inspired the discovery of Clustered regularly interspaced short palindromic repeats (CRISPR)-Cas9, which has been extensively reviewed.^42^ Later, newer defense systems, such as bacteriophage exclusion have been discovered to work by preventing DNA replication.^43^ The third potential mechanism is the abortive infection, where the infected cells are killed, and surrounding ones are protected. This mechanism is not yet fully elucidated, and still needs further exploration.

Besides the gut, the vaginal microbiota also plays crucial in resisting the colonization of invading pathogenic microbiomes, which is important for preventing sexually transmitted infections, urinary tract infections and vulvovaginal candidiasis.^44^ Traditionally, the cultivation methods suggested the vaginal microbiota as a community that lacks species that produce lactic acid (e.g., *Lactobacillus* species).^45^ Moreover, the vaginal microbial community is overabundant with anaerobic bacteria including *Gardnerella vaginalis*, *Prevotella* spp., *Mobiluncus* spp., *Ureaplasma urealyticum*, and *Mycoplasma hominis.*^46^ Later studies identified *Lactobacilli* as important members of vaginal microbiota. To better understand the vaginal microbiota, researchers have grouped the vaginal bacteria community into five types known as community state types (CSTs) I–V. All five communities are dominated by *L. crispatus*, *L. gasseri*, *L. iners*, polymicrobial flora including *Lactobacillus* and bacterial vaginosis-associated bacteria (BVAB), and *L. jensenii*. The CST I, III and IV are commonly found in women and have been extensively studied, while the other two types are rare.^47^ The *Lactobacillus* species are believed to provide protective functions by generating bactericidal and virucidal agents, including lactic acid and bacteriocins.^48^ As a result, the vaginal *Lactobacilli* is considered a risk factor of sexually transmitted infections such as human immunodeficiency virus (HIV),^49^ human papillomavirus,^50^ and herpes simplex virus infections.^51^ In a previous randomized clinical study, Schwebke et al. found that women treated with atypical gram positive stain smears showed lower risk for incident chlamydial genital infection.^52^ In another study which involves 3620 nonpregnant women, Brotman et al. found a strong association between bacterial vaginosis and elevated risk of genital infection.^53^ So far, only limited studies are available regarding vaginal colonization resistance, but it has been widely agreed upon that the vaginal colonization resistance plays crucial protective roles in preventing pathogenic infections.

The skin, as the largest organ in human body, is colonized by dense microbiome communities. Healthy skin with balanced microbiota is believed to contribute to colonization resistance against pathogenic infections. Changes in the skin microbiota (dybiosis) are highly associated with many common skin diseases, such as acne, a chronic inflammatory skin condition mediated by *Propionibacterium acnes.*^54^ Severity of P. acnes pathophysiology is correlated with the level of sebum secretion. As a result, acne is prevalent in teenager and a minor portion of adults. Also, the production of bacteriocins by current residing microbiome provides further protection against invading species.^55^ For example, *S. epidermidis* was suggested to destroy *S. aureus* biofilms via a serine protease.^56^ In addition, *S. lugdunensis* was discovered to produce lugdunin, an inhibitor of nasal colonization with *S. aureus*. Lugdunin also inhibits other pathogens including *Enterococcus faecalis*, *Listeria monocytogenes*, *Streptococcus pneumoniae*, and *Pseudomonas aeruginosa.*^57^ Overall, understanding the interactions among skin microbiota communities will be beneficial to the control of skin diseases or disorders.

### The microbiota–gut–brain axis

**Fig. 2:**
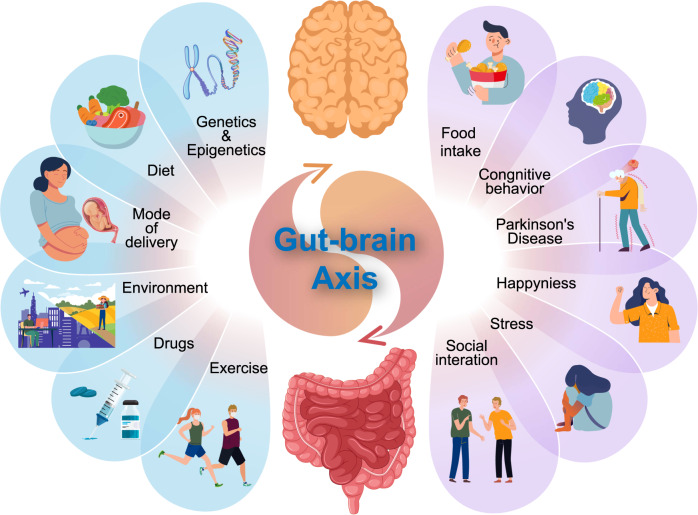
Bidirectional gut-brain axis interactions and the common factors contributing to the gut–brain activity

In the 1980s, with the development of brain imaging, our understanding of the critical roles of the gut–brain axis in homeostasis was established.^58^ Researchers then reached consensus that this axis is bidirectional. On the one hand, gut distension activates key pathways within the brain, while on the other hand, such pathways are involved with gut disorders, for example irritable bowel syndrome (IBS).^59^ In the past decades, gut microbiota was identified as a key regulator of the gut–brain axis. Multiple animal models as well as human studies have been used to model the gut-brain axis. The factors contributing to gut–brain axis balance are summarized in Fig. 2. A recent study by Chen et al.^60^ found that, due to the loss of histone demethylases (eg, KDM5), fruit flies (*Drosophila melanogaster*) showed intestinal barrier dysfunction and change in social behaviors such as mating. This is one of the direct pieces of evidence that mating behavior is likely relevant to the enteric bacteria. Similarly, in mouse models, Bravo et al. performed chronic feeding with lactic acid bacteria *Lactobacillus rhamnosus* on mice and found region-dependent alterations in the brain such as GABA gene upregulation in cortical regions and downregulation in the hippocampus, amygdala, and *locus coeruleus.*^61^ Thus, it indicates that gut microbiota could influence neurophysiology and behavior. Moreover, Buffington et al.^62^ reported that maternal high-fat diet induces gut microbiota shifts and physiological change in the offspring brain, such as fewer oxytocin immunoreactive neurons in the hypothalamus. Additionally, offspring social deficits and gut microbiota shifts could be prevented by co-housing with offspring of regular-diet mothers.^62^ This finding further supports that gut microbiota negatively impacts offspring social behavior. Gut microbiota also affects the wound-healing process. Mice fed with lactic acid bacteria *Lactobacillus reuteri* showed enhanced wound-healing properties via upregulation of oxytocin, which is a regulatory factor that activates host CD4 + Foxp3 + CD25 + immune T regulatory cells.^63^ Other studies also showed that gut microbiota impacts cognition, anxiety, depression-related behavior, and reward/addiction pathways of mice.^64^ Studies in chimpanzees (*Pan troglodytes*) revealed the other direction of microbiota in the gut-brain axis: composition of gut microbiota is impacted by various social interactions.^65^ Studies of gut–brain axis in humans showed similar results regarding the connection between brain physiology and gut microbial ecology. In 2016, Allen et al. performed a preclinical study on healthy volunteers to test if psychobiotic consumption could affect neurophysiological responses such as stress response, cognition, and brain activity.^66^ Results indicated that consumption of *B. longum* 1714 is associated with reduced stress and improved memory. However, in this study, the number of samples was small (*N* = 11) and confounding factors such as the environment, diet, lifestyles, and genetic variations were not fully considered. In another study using mouse models, gut microbiota was discovered to be necessary for motor deficits, microglia activation, and αSyn pathology.^67^ The authors transplanted the microbiota from Parkinson’s disease patients and found that the mice showed enhanced physical impairments compared with mice with microbiota from healthy donors. Thus, it suggests that gut microbes are potentially relevant to neurodegenerative diseases such as Parkinson’s disease and could be used as a therapeutic marker. Furthermore, researchers found there is significant difference in the component of microbes in the gut of children with and without autism spectrum disorders, a pervasive developmental disorder characterized by social abnormalities, communication impairments, and repetitive behaviors.^68^ This is indeed another evidence showing the relationship between GI microbiota and neurophysiology.

Many pathways have been proposed to mediate the communication within the gut-brain-axis. The signal passage along gut-brain-axis involves the interactions among autonomic nervous system (ANS), enteric neural system (ENS), central nervous system (CNS), immune system, and endocrine system. The ANS, which controls GI tract functions such as gut movement and mucus production, is a complex network that integrates the communication between the gut and the brain, as well as induces CNS effects in the gut since CNS is responsible for processing the visceral information.^69^ The ANS directly triggers neurological responses in the gut which further causes physiological changes. The ANS also mediates the interaction between the gut microbiota and ENS. ANS-triggered ENS activity results in the absorption and delivery of pre-/probiotics in the GI tract such as starches and other microbial nutrients.^70^ Microbes could affect the neural system via neuromodulatory metabolites including tryptophan, serotonins, GABA and catecholamines.^58^ Previous study on mice models have proved that gut microbial metabolite 4-ethylphenylsulfate induces mental disorders (such as anxiety-like behavior).^71^ Additionally, the gut microbial tryptophan metabolite indole was found relevant to the activation of the vagus nerve, the 10th cranial nerve that connects the gut and brain.^72^ In this study, rats with acute and high indole overproduction showed decreased motor activity, while rats with chronic and moderate indole increase showed enhanced anxiety-like behavior. Similarly, the bacteria *Lactobacillus rhamnosus* was found to induce information transmission in vagal afferents in the mesenteric nerve bundle. Such induction could be eliminated by vagotomy.^73^ Also, treatment with bacteria *Lactobacillus reuteri* in rats models was found to help mice with social deficits; such change was also restored in mice with vagotomy.^74^ Recent studies also reported potential mechanisms of microbiota–ENS interaction. As one of the major serotonin producers in human, gut microbiota is linked to ENS activation by 5-HT receptors. De Vadder et al. demonstrated the interaction by pharmacological modulation of 5-HT receptors and depletion of endogenous 5-HT.^75^ The presence of 5-HT receptor antagonist negatively affects ENS activity. The gut microbiota also communicates with another major neuroendocrine system, the hypothalamic–pituitary–adrenal (HPA) axis, which is known to coordinate stress response.^76^ Signal molecules generated in HPA are distributed throughout the body and affect gut microbiota. To illustrate the connection between HPA and gut microbiota, GF mice are used. Studies revealed that GF mice exhibited elevated plasma corticosterone, indicating hyperresponsive HPA axis and the regulatory effect of gut microbiota.^77^ In human, it has been reported that bowel syndrome patients (with gut microbiota changes) tend to have exaggerated adrenocorticotrophic hormone response to corticotrophin releasing factor infusion.^78^ Despite there have been numerous of studies on the bidirectional pathways between the gut-microbiota and the brain, it is still difficult to fully understand the mechanisms.

Numerous influencing internal and external factors have been discovered to modulate the gut-brain axis of the host, including genetics, socioeconomic status, diet, medications, and environmental factors.^79^ Genetics and epigenetics are important in understanding the brain as well as gut health. An increasing number of studies have been performed on the relationship between host (human or mice) and microbiota genetics. One of the important components of microbiota-host genetic interaction is via the modulation of RNAs. For example, in GF mice models, researchers found that microRNAs were dysregulated in GF mice in certain brain regions, amygdala and prefrontal cortex, which suggests a close relationship between gut microbiota and brain physiology.^80^ In another study of gut microbiota and hippocampal RNAs using GF mice, Chen et al. found that gut microbiota significantly regulates the expression level of hippocampal microRNAs and mRNAs. Specifically, re-colonizing the gut microbiota in GF mice did not reverse the behavioral change such as less latency to familiar food, but microRNAs and mRNAs were significantly restored.^81^

As mentioned before, lifestyles, especially diet, have been shown to be among the most critical factors in modulating the gut-brain axis. For example, a high-fat diet with only animal products will shift the microbiota composition profoundly. Specifically, animal models with high-fat diet showed reduction in *Bacteroidetes* levels and an increase in both *Proteobacteria* and *Firmicute* levels.^82,83^ *Proteobacteria* (*Bilophila wadsworthia*) abundance was also observed in another study of high-fat diet-fed animals.^84^ On the contrary, the Mediterranean diet composed of whole grains, nuts, vegetables, fruits, and only certain animal products (fish and poultry) showed beneficial results in hosts. In human intervention studies of diet, consumption of the Mediterranean diet has been shown to significantly reduce the occurrence of neurovegetative disorders, psychiatric conditions, cancer, and cardiovascular diseases.^85^ Mediterranean diet also showed correlation with reduced risk of depression.^86^ Though strong evidence showed that the Mediterranean diet is beneficial to the hosts, further mechanism studies are still necessary to illustrate the regulatory mechanism of such diet on the gut–brain axis. Another type of diet with high fat and low carbohydrate, namely the ketogenic diet, is popular because it forces the consumption of the body’s reserved fat. Ketogenic diet was considered to be able to inhibit apoptosis in neurodegenerative diseases because of the increase in serum ketones, which has been shown to improve mitochondrial activity.^87^ Studies have shown that the ketogenic diet also causes shift in microbiota abundance in the gut. Specifically, *Akkermansia*, *Parabacteroides*, *Sutterella*, and *Erysipelotrichaceae* levels were significantly higher in mice on ketogenic diets.^88^ Moreover, mice on ketogenic diets were better protected from acute epileptogenic seizures compared with the control group on a normal diet. Furthermore, colonization with increased microbiomes in GF mice also showed correlation with seizure protection, as well as alterations in hippocampal metabolomic profiles. All above studies support the conclusion that changes in lifestyles have marked impacts on the gut microbiota.

Finally, medications, especially antibiotics, will directly affect the gut microbiota and subsequently the gut–brain axis. Besides antibiotics, a growing number of studies also showed that nonantibiotic drugs can change the gut–microbiota composition, as well as neurophysiology and behavior.^89^ In a large-scale gut-microbiota project named the Belgian Flemish Gut Flora Project, antibiotics, osmotic laxatives, hormones, benzodiazepines, antidepressants, antihistamines, and inflammatory bowel disease drugs were found to be highly relevant to the variation of gut microbiota.^90^ Other studies also showed that proton pump inhibitors, metformin, and statins can impact gut microbiota.^91^ Moreover, due to the rise of interest in the gut–brain axis, more and more psychotropic medications were discovered to have antimicrobial activities. Examples are serotonin antagonists such as sertraline, paroxetine, and fluoxetine, which have antimicrobial activity against gram-positive bacteria such as *Staphylococcus* and *Enterococcus.*^92^ These findings indicate the potential impact of medications on the gut–brain axis.

### Microbiota in the development of immune systems

Microbiota in different organs exhibits distinct characteristics and compositions. As a result, microbiota interacts with multiple biological processes of the host. In this section, we introduce the interactions between human microbiota in gut, oral cavities, lungs, skin, vagina, and the development of immune systems.

The human immune system is comprised of innate and adaptive immune responses, both of which have been shown to extensively interact with microbiota. The innate immune response has critical role in maintaining a homeostatic environment by eliminating pathogenic bacteria and regulating the adaptive response to microbiota. These effects are mediated by factors such as secretory IgA (sIgA), toll-like receptor 5 (TLR5), autophagy, and inflammasomes.^93^ For instance, slgA can bind and form complexes with commensal bacteria, which selectively presents the bacterial components to tolerogenic dendritic cells. As an anti-inflammatory molecule, slgA can reduce the inflammatory response that could result from the immense bacteria load in the organs. On the other hand, dysbiosis of microbiota can alter the sIgA response and lead to unregulated bacterial growth. Hapfelmeier et al. showed that microbiota-specific sIgA response was observed in GF mice using reversible microbial colonization system.^94^ The sIgA induction was confirmed as a gradual response to current bacterial exposure, suggesting a crosstalk between microbiota and immune system. The adaptive immune response is another important part to maintain a healthy microbiota and immune balance. Particularly, the education of adaptive immune response is achieved by differentiation and maturation of B and T cells and establishment of immune tolerance to microbiota.^95^ Depending on the bacteria species, the CD4 T cell responses vary significantly, which leads to the differentiation into distinct subsets and the subsequent pro-inflammatory cytokine release such as interferon-γ and interleukin IL-17A. The crosstalk between microbiota and adaptive immune response will be further discussed in the following sections.

**Fig. 3:**
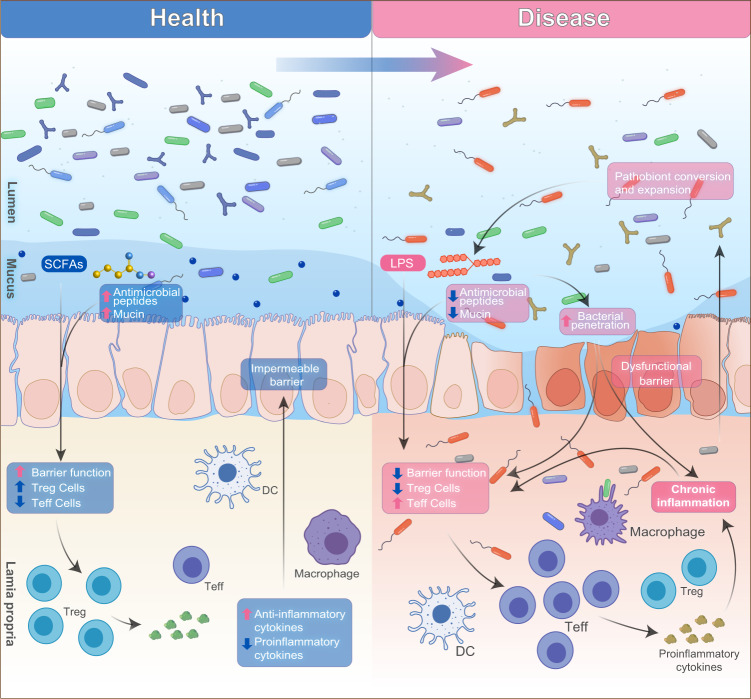
Factors affecting microbiota-associated chronic inflammation in healthy and disease state

The GI tract hosts a large number of immune cells, which constantly communicate with the gut microbiota. The maturation of the immune system needs the development of commensal microorganism. One of the mechanisms of gut microbiota affecting the immune system is by mediating neutrophil migration, which subsequently impacts T cell differentiation into various types such as helper T cells (Th1, Th2, and Th17) and regulatory T cells.^96^ Disorders in microbiota development during the maturation of the immune system could lead to deteriorated immunological tolerance and autoimmune diseases.^97^ Additionally, heterogeneous molecules produced by microbiota may induce immune response and stimulate inflammation or chronic tissue damage.^98^ The general interactions of microbiota and host immune response during healthy and disease states are depicted in Fig. 3.

Human immune system is closely related to the microbiota as a complex symbolic relationship during the co-evolution of vertebrates and microbiota.^99^ The vertical transmission from the mother’s microbiota to the child at birth is considered the initial introduction of microbiota to the child. As a result, infants born by Cesarean section are colonized with bacteria of the epidermal origin, which might link to higher risk of developing allergies and asthma compared with infants who received initial microbiota from the maternal vaginal flora.^100^ Such difference in immune system and microbiota would be gradually eliminated during growth. As mentioned before, the infant’s microbiota stabilizes at ~1-year-old and resembles that of adults. The neonatal immune system also rapidly develops under the impact of dynamic microbiota.^101^ In addition to the microbiota transmission during birth, breastfeeding also plays crucial roles in the establishment of infant immune system as well as microbiota. Besides the required nutrients and antimicrobial proteins, breast milk provides slgA, which is specifically shaped by the maternal microbiota. As a result, infants’ microbiota is seeded not only by the maternal epidermal or virginal origin but reinforced by the sIgA shaped by maternal microbiota. Moreover, before the solidification of infant immune system, the sIgA significantly protects the newborn against pathogens.^99^ To summarize, the maternal-neonatal microbial bond supports the close relationship between microbiota and the immune system.

The gut microbiota has been closely connected to immunological response due to the fact that enteric microorganisms may promote macromolecules and antigens through the gut epithelium.^102^ The principal component of bacterial flagellum, namely flagellin, elaborates the relationship between gut epithelial integrity and host immunity. Flagellin is recognized by TLR5, which is found actively expressed in B-cells and CD4 + T-cells. Differentiated B-cells produce IgA, which neutralizes the pathogen and potential subsequent infection.^103^

The gut microbiota contributes to the development of immune system via the gut-associated lymphoid tissues composed of Peyer’s patches (PPs), plasma cells, and lymphocytes. Previous studies have shown that the gut bacteria interact with mucosal antibodies that are taken up by CD11 + dendritic cells in the PPs. Studies also showed that the luminal microbiota bound to SIgA increased their presence in PPs.^104^ The CD8 + T cells are mostly found in the intraepithelial intestine compartment, and the microbiota plays important roles in maintaining the function of CD8 + T-cells. This is supported by previous studies showing that GF mice exhibit reduced intestinal CD8 + T-cells.^105^ In all, understanding the relationship between the microbiota and the immune system is a critically important topic in health sciences. However, due to our innate understanding of the network of gut-immune system, greater attention will be necessary to further promote our knowledge in immune homeostasis and novel immune-microbe therapies.

The oral cavity is another important habitat where the microbiota could colonize. Different from the gut environment, the oral cavity contains both hard surface of teeth and epithelial surface of mucosal membrane. Approximately 50 species (1000 sub-species) exist in the oral microbiota. Due to the constant exposure to saliva, oral microbiota acquired the feature of avid adherence, which guarantees their colonization and resistance to the forces of fluid flow.^106^ Oral microbiota contains complex polymicrobial communities which have complicated interactions with the host’s diet and immunity. The colonization resistance in oral microbiota is affected by not only the lack of a single treatment for therapeutic intervention, but also due to the presence of a fluid phase which could inactivate bioactive molecules. The number of different oral sites where disease can occur and the poor retention of topical application of therapies are also hurdles to the treatment of oral disease caused by pathogens. Oral pathogens exert the ability to trigger immune response such as pro-inflammatory responses. On the other hand, alterations in host immune system also affects the oral microbiota community. For example, gingivitis, a common disease in humans, is caused by immune-inflammatory responses where neutrophils are recruited to the gingival tissues.^107^ In periodontal disease, inflammation has been found to be an important driver for the growth of pathogenic microorganisms since inflammation can cause tissue destruction, which provides nutrient to microbiomes.^108^ However, inflammation could subsequently trigger bactericidal activity of the immune system. Thus, there exists such a paradox in dysbiosis that if the host immune system was downregulated, microbiomes will starve due to lack of nutrients. Periodontitis-associated bacteria such as the *P. gingivalis* is able to tackle the conundrum by triggering the host immune response without coupling bactericidal activity. Such function has been demonstrated in mice models where *P. gingivalis* intervened the host-microbiota homeostasis and contributed to the development of periodontitis.^109^ The special manipulation of the host immune system by *P. gingivalis* has been revealed to involve C5a receptor 1 (C5aR1) and TLR2. In human and mouse neutrophils, *P. gingivalis* was able to initialize a C5aR1-TLR2 signal which separates a TLR2-MyD88 pathway from a TLR2-MyD88-Mal-PI3K pathway, leading to inflammation and blocked phagocytosis. In summary, the oral microbiota could be both beneficial by potentially stabilizing the microbial diversity and harmful to cause collective pathogenic outcomes.

Like gut and oral tissues, the lungs also present a complex bacteria community. The lung microbiota is relatively dynamic as a result of the microbiome immigration and elimination via aspiration, cough, or mucociliary clearance.^110^ The majority of microbes in lungs belong to *Bacteroidetes*, *Firmicutes*, *Proteobacteria*, and *Actinobacteria* families.^111^ The lung microbiota is responsible for the state of immune tolerance that protects the host from undesired inflammatory response.^112^ This function is mediated by the interaction between commensal bacteria and lung immune cells. Given the important role that lung microbiota plays in maintaining lung homeostasis, the lung microbiota composition is useful in monitoring lung health conditions.^113^ The interactions between lung microbiota and local immune cells are closely relevant to the pattern recognition receptors (PRRs), which are responsible for the recognition of microbial molecules. The above-mentioned TLRs also belong to PRRs. Activation of PRRs could stimulate the engagement of ligands and further induces immune-related genes expression, which promotes the immune response against pathogens.^114^ Additionally, the lung microbiota was also found to regulate antigen-presenting cells and regulatory T cells. In mice models, it was found that newborn mice showed excessive airway eosinophilia, Th2 cytokine release, and hyper-responsiveness after exposure to allergens. With the bacterial load increasing during the following two weeks, the microbiota composition was shifted (*Gammaproteobacteria* and *Firmicutes* toward *Bacteroidetes*) and responsiveness to allergens was decreased. The major mechanism includes the appearance of the Helios-regulatory T cells subset, which is promoted by changes in lung commensal bacteria community.^115^ Furthermore, infant mice without proper lung microbiota would suffer from excessive sensitivity to allergens until adulthood.

The human skin, like gut, is also colonized by a dense community of microbiomes composed of highly diverse communities. It has been discovered that the skin microbiota is composed of prokaryotes (bacteria and archaea) and eukaryotes (fungi, metazoic parasites). Similar to the gut microbiota, skin microbiota is also involved in the development of the innate immune system. For example, *S. epidermidis* produces lipoteichoic acids which prevent skin from injury-induced inflammation. The potential mechanisms include the inhibition of cytokine release and TLR2-based immune responses.^116^ Interestingly, *S. epidermidis* also promotes the expression of certain antimicrobial peptides like human β-defensins (hBDs) which enhances the skin defense.^117^ Moreover, *S. epidermidis* was believed to strengthen the function of skin lymphocytes, thereby contributes to increased skin immunity.^118^ In summary, as a primary part of the human immune system, the skin harbors a wide range of cells that perform functions of immunity such as macrophages, dendritic cells, lymphocytes and various T-cell populations. Moreover, due to the advent of high-throughput sequencing, researchers are able to perform in-depth taxonomic analysis of the skin microbiota, which further boosts our understanding of roles that the skin microbiota plays in human wellness.

As mentioned above, vaginal microbiota is critical in protecting the host from invading pathogens via colonization resistance. It was also revealed that vaginal microbiota drives the innate immune response. Specifically, the vaginal microbiota stimulates the PRRs in and on epithelial cells lining the vagina and upper genital tract and initializes cytokine signaling cascades.^119^ For example, the release of interleukin (IL)-1β/6/8 and Tumour Necrosis Factor alpha (TNF-α) recruits or activates immune cells like Natural killer (NK) cells, macrophages, CD4 + helper T-cells, CD8 + cytotoxic T-lymphocytes and B-lymphocytes.^120^ Bacterial vaginosis (BV) is one of the most common vaginal dysbiosis due to the displacement of *Lactobacillus spp* and the increased concentration of BVAB. Pathogenic microbiomes in BV such as *G. vaginalis* and *P. bivia* have been found to inhibit the host inflammatory response in the vaginal epithelium.^121^ However, only limited number of studies examine the mechanisms of how BVAB interacts with the host immune system. Previous studies revealed that *G. vaginalis* infection does not trigger changes in the level of pro-inflammatory mediators including IL-1β, IL-6, MIP-3α, or TNFα,^122^ while *A. vaginae* induces a broad range of pro-inflammatory cytokines, chemokines, and antimicrobial peptides including IL-1β, IL-6, IL-8, MIP-3α, TNFα, and hBD-2; whereas *P. bivia* induces fewer types of immune factors including IL-1β and macrophage inflammatory protein (MIP)-3α.^123^ However, contradictory results have been reported that *P. bivia* suppressed the host immune responses.^124^ In all, further studies are still necessary to better understand the interaction between vaginal microbiota and host immune system.

## Microbiota in the development of diseases

**Fig. 4:**
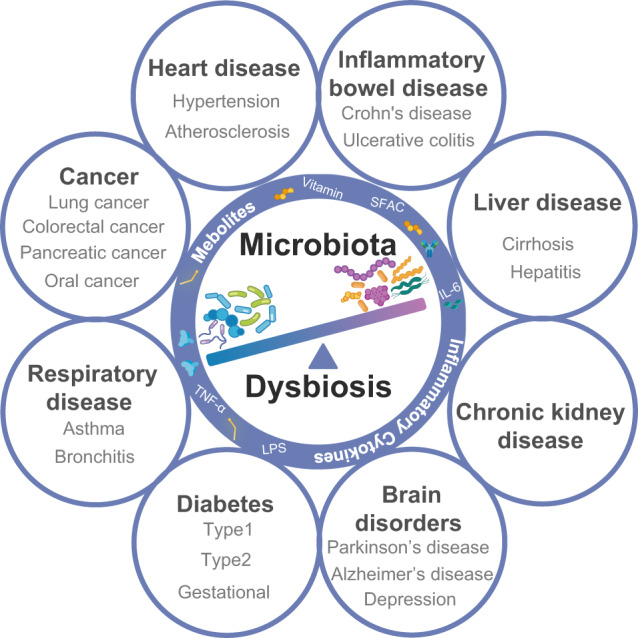
Human microbiota dysbiosis contributes to various diseases

Microbiota are complex systems consisting of trillions of microorganisms. With advanced sequencing technologies and bioinformatics, most of microbiota–related research is focusing on the relationship between microbiota compositional changes and various disease states. When subjected to external changes, the balance of microbiota community can be affected, leading to dysregulation of bodily functions and diseases^125^ as summarized in Fig. 4. To date, mounting evidence has confirmed that microbiota is associated with the development of CVDs, cancer, respiratory diseases, diabetes, IBD, brain disorders, chronic kidney diseases, and liver diseases. Due to the limited studies on non-bacterial species in disease development, we majorly focus on the bacterial element of the microbiota in this section. The disease-related pathogens and the signaling pathways are summarized in Table 2 and are discussed in detail in each section.

### Cardiovascular diseases

CVDs are the leading cause of morbidity and mortality worldwide, including coronary heart disease, cerebrovascular disease, peripheral arterial disease, etc. While the general risk factors include atherosclerosis, hypertension, obesity, diabetes, dyslipidemia, and mental illness, growing evidence has suggested that microbiota play a role in maintaining cardiovascular health and its dysregulation may contribute to CVDs.^126^ Particularly, studies on microbiota transplantation, microbiota-dependent pathways, and downstream metabolites have all shown that microbiota may influence host metabolism and CVDs through multiple metaorganism pathways. Here, we present the potential pathogenesis of microbiota in CVDs.

#### Oral microbiota

Periodontal diseases, which are initiated and propagated through dysbiosis of oral microbiota, have been shown to be associated with increased risk for CVDs. In 1993, DeStefano et al. reported that subjects with periodontitis had a 25% increased risk of CVDs compared with those with minimal periodontal disease, indicating a correlation between oral microbiota with CVDs.^127^ Multiple bacterial phylotypes were found in both the oral cavity and atherosclerotic plaques, suggesting an association between oral microbiota and atherosclerosis. Schenkein et al. presented two major mechanisms linking periodontitis with atherosclerosis.^128^ The first is that some microorganisms can invade endothelial and phagocytic cells within the atheroma, causing pathogenic changes and progression of the lesion, and the second includes the release of inflammatory mediators such as C-reactive protein (CRP), fibrinogen, metalloproteinases from periodontal lesions to systemic circulation. A cohort study by Lise et al. reported that the antibody levels to periodontopathogen *T. forsythia* were inversely related to the increased risk for CVD mortality. Indeed, other studies also linked periodontitis with several cardiovascular risk factors. A randomized controlled trial (RCT) showed that intensive periodontal treatment achieved a reduction in systemic inflammatory markers including IL-6 and CRP, and a decreased systolic blood pressure and an improvement in lipid profiles.^129^ IL-6 can cause cardiac hypertrophy through the IL-6 signal-transducing receptor component, glycoprotein 130. Moreover, IL-6 is able to induce the hepatic synthesis of CRP. CRP is suggested to directly influence vascular vulnerability through several mechanisms including regulating the local expression of adhesion molecules, downregulating the endothelial bioactivity of nitric oxide, altering the low-density lipoprotein ingestion of macrophages. Ramirez et al. found that, compared with the control group, periodontitis patients had higher levels of E-selectin, myeloperoxidase, and ICAM-1, which are important risk markers for CVDs.^130^ E-selectin is a receptor of carbohydrate ligands on the surface of leukocytes. It functions by binding with leukocytes, drawing them from the circulation toward the surface of the endothelium. Subsequently, the transmembrane glycoproteins ICAM-1 and VCAM-1, interact with integrins on the surface of the leukocyte to promote its strong binding to the endothelium, thereby contributing to CVD through their inflammatory effects on the vascular endothelium.

#### Gut microbiota

It is not surprising that the gut microbiota, is considered the largest endocrine organ in the body, can affect the cardiovascular system and contribute to CVDs. Gut microbiota is involved in the metabolism of choline, phosphatidylcholine, and carnitine, which eventually produce trimethylamine-N-oxide (TMAO). TMAO has been suggested to not only regulate cholesterol balance and bile acid levels but is also associated with early atherosclerosis and high long-term mortality risk of CVDs.^131^ Mechanistically, TMAO can activate the mitogen-activated protein kinase (MAPK) and NF-κB signaling pathways in endothelial cells and smooth muscle cells.^132^ The MAPK signaling pathway can be stimulated by growth factors, pathogen-associated molecules, and inflammatory cytokines, which follows by a MAPKKK-MAPKK-MAPK-TFs signaling cascade and results in the expression of inflammatory cytokines IL-6, IL-8, and TNF-α. It is well-established that NF-κB is an important mediator that regulates the activation, differentiation, and effector function of inflammatory immune cells. Therefore, the dysregulation of NF-κB may contribute to pathogenesis of atherosclerosis by promoting monocyte recruitment.^133^ Another inflammation mediator lipopolysaccharide (LPS), also known as endotoxin, is a component of Gram-negative bacteria that are mainly distributed in gut and oral cavity. Recent studies have shown that LPS can induce vascular oxidative stress by activating the TLR4 pathway, leading to endothelial dysfunction and vascular inflammation. A retrospective analysis conducted by Yoshida et al. suggested that patients with CVDs have higher fecal LPS levels compared with those without CVDs. It is interesting that the structures of lipid A moieties of LPS differ in bacteria, which may determine LPS activity.^134^

Gut microbiota is able to metabolize polysaccharides and proteins into short-chain fatty acids (SCFAs), another class of metabolites that is linked to CVDs. Most SCFAs are acetates, butyrates, or propionates. A large proportion of acetates is subjected to lipogenesis in adipose tissue and oxidize in muscle, with some being converted to butyrates by bacteria. As shown in Fig. 3, butyrates are involved in mediating the integrity of the intestinal barrier and are suggested to have direct salutary effects on intestinal epithelial cells.^135^ Propionates are mainly oxidized or metabolized in the liver. The potential role of these major SCFAs in CVDs has been extensive studied in animal models. SCFAs, particularly propionates and butyrates, were shown to protect the host from hypertensive cardiovascular damage.^136^ The propionate is suggested to regulate the balance of effector T cells and regulatory T cells, which is critically important in hypertension and hypertension-induced organ damage.^137^ Moreover, propionates reduced lateralization of gap junction protein connexin 43 in cardiomyocytes, thereby reducing susceptibility to ventricular tachycardia.^136^ The butyrate has been shown to modulate blood pressure by inhibiting expression of renal prorenin receptors and renin in animal models.^138^ Recently, accumulating evidence has shown that SCFAs can act on G-protein-coupled receptors Gpr41, Gpr43, and Olfr78 to mediate blood pressure. Olfr78, expressed in smooth muscle cells of vasculature, is an olfactory receptor that mediates renin secretion in response to SCFAs. Gpr41 and Gpr43 are widely expressed in the body, which will be activated upon SCFAs binding. It is established that Olfr78 and Gpr41/43 response to SCFAs through different G protein subunits and second-messenger systems. Olfr78 will activate adenylate cyclase type 3 and G_olf_ in the olfactory signaling pathway to induce cAMP production; while Gpr41/43 activates Gαi and/or Gαo to decrease cAMP.^139^ Therefore, activation of Olfr78 increases hypertension by facilitating the release of renin, while activation of Gpr41 and Gpr43 counteract the hypertensive effect of Olfr78.^140^ These data reinforce the important role of microbiota in blood pressure control and CVD progression.

### Cancer

Cancer is a disease characterized by the rapid proliferation of abnormal cells that grow uncontrollably, which can occur in almost all regions of the body. Currently, cancer is a leading cause of mortality worldwide, causing over 10 million deaths in 2020.^141^ Generally, the development of cancer is due to gene mutations that disrupt the cell growth or metabolic activities, and more than 100 human carcinogens are listed by the World Health Organization. Although carcinogenesis is a multifactorial process, it has been well established that tobacco, bacteria and viruses, obesity, alcohol, and radiation are the major risk factors for cancer.^142^ While the role of microorganisms was disregarded in cancer for a long time, the focus has shifted largely due to the finding that *H. Plyori* contributes to gastric cancer initiation in 1994.^143^ Surprisingly, recent studies have shown that microbiota plays an important role in carcinogenesis, mainly through 1) influencing the host cell proliferation and death, 2) altering immune system activity, and 3) affecting host metabolism.

#### Oral microbiota

Researchers have found that periodontitis, characterized by dysbiosis of oral microbiota, is involved in the initiation and progression of oral, pancreatic, genitourinary, and gastrointestinal cancers. Farrell et al. found a significant variation between the salivary microbiota of pancreatic cancer patients and healthy subjects in a retrospective case–control study.^144^ In cancer patients, the levels of *N. elongate*, and *S. mitis* were significantly decreased, while the level of *G. adiacens* was elevated compared with healthy subjects. A prospective cohort study conducted by Michaud et al. revealed that individuals with high *P. gingivalis* antibody levels had a twofold increased risk of pancreatic cancer compared with those with low antibody levels.^145^ Similarly, Fan et al. suggested that *P. gingivalis* was correlated with higher risk of pancreatic cancer, while *Fusobacteria* were associated with a decreased risk.^146^ Studies revealed that the abundance of *T. forsythia*, *P. gingivalis*, and *F. nucleatum* are significantly higher in esophageal cancer tissues compared with normal tissues.^147^ It is suggested that oral microbiota promote carcinogenesis by inducing chronic inflammation and producing oncometabolites.^148^ Since many bacteria share similar carcinogenic mechanisms, we use *P. gingivalis*, a pivotal periodontal bacterium, as an example to introduce the pathogenesis. Oral squamous cell carcinoma (OSCC) is the most common cancer in the head and neck region. Firstly, the presence of *P. gingivalis* has been shown to increase the risk of OSCC by dysregulating tissue integrity and host immune response. Cao et al. suggested that *P. gingivalis* can bind to TLR4 receptor, which in turn activate NF-κB pathway and overstimulate the downstream JAK1/STAT3 signaling pathway, leading to inhibition of cell apoptosis.^149^ TLRs are characterized as primary sensors that response to microbial components and trigger immune response. All TLR signaling pathways eventually activate NF-κB pathway, which controls the expression of a wide range of inflammatory cytokines. Secondly, studies have shown that *P. gingivalis* can stimulate the proliferation of epithelial cells by interfering with the cell cycle regulation. Kuboniwa et al. reported that *P. gingivalis* can affect signaling pathways involving cyclins, p53, and PI3K.^150^ Cyclins are subunits of CDK complexes that regulate the progression of cell cycle and thus proliferation. p53 is a tumor suppressor gene that has been well-established in the cause of cancer. Activation of p53 can cause cell cycle arrest and apoptosis, thus mutation or loss-of-function of p53 may lead to uncontrolled cell growth.^151^ Moreover, *P. gingivalis* has been shown to interact with β-catenin, a key protein in regulating cell proliferation and tumorigenesis. The Wnt/β-catenin signaling is a versatile pathway that involved in many human diseases. Aberrant activation of Wnt/β-catenin pathway results in the accumulation of β-catenin in the cells and thus upregulating the expression of oncogenes including CyclinD-1 and c-Myc. Zhou et al. suggested that *P. gingivalis* can induce noncanonical activation of β-catenin and dissociation of the β-catenin destruction complex via gingipain-dependent proteolytic processing.^152^ Thirdly, *P. gingivalis* may induce chronic inflammation by increasing levels of cytokines including IL-8, TGF-β1, and TNF-α. IL-8 and TGF-β1 can enhance the invasiveness of tumor cells by upregulating matrix metalloproteinases.^153^ TNF-α can lead to gene mutations through the generation of reactive oxygen species (ROS) or reactive nitrogen (RNS) intermediates as well as induce epithelial–mesenchymal transition, which stimulates tumor angiogenesis.^154^ Lastly, *P. gingivalis* can produce oncometabolites such as acetaldehydes and oxygen radicals. Accumulation of these metabolites are known to promote chronic inflammation and cause DNA damage and mutagenesis, leading to cancer development. Recent studies also suggested that intestinal colonization of oral microbiota contributes to several health issues including carcinogenesis.^155^ *F. nucleatum*, a periodontal pathogen, has been extensively studied in colorectal cancer (CRC). By comparing cancer and adjacent normal tissues, it was found that *F. nucleatum* was significantly enriched in tumor tissues and may promote CRC progression by increasing tumor multiplicity and selectively recruiting tumor-infiltrating myeloid cells.^156^

#### Respiratory microbiota

The focus of respiratory microbiota and cancer is largely on lung cancer. The lung, which was once considered sterile, is colonized by different microbiota throughout the respiratory tract. In healthy individuals, the core microorganisms in the lung are *Pseudomonas*, *Streptococcus*, *Prevotella*, *Fusobacterium*, *Haemophilus*, *Veillonella*, and *Porphyromonas*. In a systematic review, Perrone et al. summarized that levels of *Actinomyces*, *Veillonella*, *Streptococcus*, *Megasphaera*, and *Mycobacterium* were more abundant in lung cancer patients compared with healthy individuals. In addition, Gomes et al. reported that a squamous cell carcinoma subcluster with the worst survival was correlated with several Enterobacteriaceae.^157^ Another study suggests that in the microbiota of patients with lung cancer, unlike in the control group, has high levels of Streptococcus, indicating it may be a possible diagnostic marker. Interestingly, Peter et al. found no relationship between tumor tissue microbiota with lung cancer recurrence, while the higher richness and diversity in adjacent normal tissue was associated with worse outcome.^158^ Although the underlying carcinogenesis mechanisms are not fully elucidated, dysbiosis of lung microbiota increases inflammation and host immune modulation, which are two important pathways related to cancer. Jin et al. reported that microbiota induced inflammation associated with lung adenocarcinoma via activation of lung-resident γδ T cells, facilitating the proliferation of tumor cells.^159^ Interestingly, previous studies suggested that γδ T cells are able to recognize cancer cells and initiate anticancer activity, largely related to cytotoxicity and interferon-γ production. Therefore, it remains inconclusive how the immune system response to lung microbial. In an epithelial cell model, exposure to *Streptococcus*, *Prevotella*, and *Veillonella* led to the upregulation of PI3K and ERK1/2 signaling pathways, which mediates cell proliferation, differentiation, and survival.^160^ PI3K/Akt/mTOR is one of the most important cell signaling pathways and a well-established mediator of cancer. Activation of PI3K/Akt/mTOR pathway, by gene mutation, inactivation of PTEN, or activation of upstream oncogenes, contributes to the development of tumor and resistance to therapeutics. The same research group used an in vivo non-small cell lung cancer mouse model to show that microbiota dysbiosis led to upregulation of PI3K/AKT, ERK/MAPK, IL17A, IL6/8, and inflammasome pathways, suggesting that microbiota can contribute to the pathogenesis of lung cancer.^161^

#### Gut microbiota

Increasing evidence suggests that gut microbiota is associated with the initiation and progression of CRC. Studies have shown that dysbiosis of gut microbiota can trigger inflammation and immune response that are indirectly related to carcinogenesis.^162^ Grivennikov et al. reported that microbial products may induce epithelial barrier deterioration, which trigger tumor-elicited inflammation and drive initiation and progression of CRC.^163^ It is suggested that gut microbiota can promote CRC progression by affecting certain signaling pathways including E-cadherin/β-catenin, TLR4/MYD88/NF-κB, and SMO/RAS/p38 MAPK.^164^ Chen et al. suggested that both commensal and pathogenic bacteria facilitate CRC progression via 1) exploiting tumor surface barrier defects, 2) invading normal colonic tissue and inducing local inflammation, and 3) producing genotoxic metabolites to induce oncogenic transformation of colonic epithelial cells.^165^ It has been characterized that the major bacteria that contribute to CRC are *E. faecalis*, *E. coli*, *B. fragilis*, *S. bovis*, *F. nucleatum*, and *H. pylori.*^166^ These bacteria are able to produce genotoxic substances such as colibactin, *B. fragilis* toxin, and typhoid toxin that cause host DNA damage. For example, intestinal *F. nucleatum* was evaluated in several CRC studies.^167^ The abundance of *F. nucleatum* was significantly higher in mucosal and fecal samples of CRC patients compared with healthy controls. These studies also indicated that *F. nucleatum* can invade CRC tumor cells, leading to the presumption that *F. nucleatum* may influence tumorigenesis.

### Diabetes mellitus

Diabetes mellitus (DM) refers to a group of diseases that affect glucose regulation. DM can be classified as type 1 diabetes mellitus (T1DM), Type 2 diabetes mellitus (T2DM), and gestational diabetes mellitus (GDM). T1DM is caused by the autoimmune response against pancreatic β cells, while T2DM is characterized as the inability of the body to produce or utilize insulin properly. GDM is one of the most prevalent pregnancy complications and is associated with increased risk of maternal and fetal metabolic disorders. The relationship between microbiota and DM has been extensively studied and the correlation between microbiota dysbiosis and onset of DM is well established.

#### Type 1 diabetes mellitus

In T1DM, the microbiota is an attractive research field due to its close relationship with chronic inflammation and immune response. The composition of oral and fecal microbiota appears to be distinct in T1DM patients in multiple studies. Groot et al. found that *Christensenella* and *Bifidobacteria* were enriched in fecal samples.^168^ Oral *Streptococcus* was positively associated with T1DM, while fecal *Streptococcus* was inversely correlated with T1DM. In addition, T1DM patients may exhibit decreased levels of SCFA butyrate-producing bacteria, which are key factors in decreasing chronic inflammation and maintaining intestinal homeostasis.^169^ This finding was reinforced by other case-control studies which showed that the levels of *R. faecis*, *F. prausnitzzi*, *Intestinimonas* were significantly lower in T1DM patients than in healthy controls.^170^ However, inconsistent results were reported with other bacteria, suggesting that further research is required.^171^ Interestingly, Vatanen et al. analyzed data from TEDDY study and showed that healthy children contained more genes related to fermentation and the synthesis of SCFAs without significant association to specific taxa.^172^ Therefore, it is possible that the T1DM-related microbial factors are taxonomically diffuse but functionally coherent. The research on microbiota in T1DM was conducted mainly in animal models, therefore, the pathogenic mechanisms require further validation in human. It was first introduced that the development of T1DM may be dependent on microbiota in 1987 by Suzuki et al.^173^ It was suggested that microbiota can contribute to T1DM mainly through immune response modulation. T1DM is defined as an autoimmune disease by chronic inflammation of the pancreatic islets of Langerhans. Since microbiota is involved in the initiation of chronic inflammation, it is not surprising that its dysregulation may contribute to T1DM. Higuchi et al. reported that the plasma levels of IL-6 were significantly higher in T1DM patients than in healthy controls, which was correlated with the abundance of *Ruminococcaceae* and *Ruminococcus*. Leiva-Gea at al also reported that T1DM patients had increased levels of proinflammatory cytokines IL-1β, IL-6, and TNF-α and decreased levels of anti-inflammatory cytokines IL-10 and IL-13, which were significantly correlated with the abundance of different bacteria.^170^ In addition, an upregulated level of LPS was observed, which is known to induce the release of proinflammatory cytokines and impair pancreatic β cells.^174^ Clinical data showed that T1DM patients have significantly elevated levels of TLR2 and TLR4 ligands, indicating increased TLR2 and TLR4 activity.^175^ As described above, TLRs play an important role in innate and adaptive immunity, which protect the body from infectious microorganisms. The role of TLR4 was further evaluated in mouse models. Elke et al. reported that TLR4 accelerates the development of diabetes, suggesting that TLR4 is involved in the progression of insulitis.^176^ The TLR4/MyD88 pathway regulates the activation of NF-κB and the levels of pro-inflammatory cytokines such as IL-6 and TNF-α.^177^ Wen et al. established an MyD88-negative Non-Obese Diabetic mice and found that the mice lacking MyD88 protein do not develop T1DM. Taken together, it suggests that microbiota may facilitate the progression of T1DM via TLR4/MyD88 signaling pathway.

#### Type 2 diabetes mellitus

In terms of T2DM, gut microbiota has been linked to disease development. Numerous studies have confirmed that the composition of gut microbiota is altered in T2DM patients.^178^ Larsen et al. reported that the abundance of *Firmicutes* and *Clostridia* were significantly decreased in T2DM patients compared with the control group. In addition, the ratios of *Bacteroidetes* to *Firmicutes*, *Bacteroides*-*Prevotella* group to *C. coccoides*-*E. rectale* group were positively correlated with blood glucose level.^179^ Almugadam et al. showed that the abundance of SCFA-producing bacteria *Facalibacterium* and *Roseburia* were significantly decreased in T2DM patients.^180^ Antidiabetic agents were able to improve the diversity and richness of gut microbiota and enriched gut ecosystem with beneficial bacteria. The underlying molecular mechanisms of gut microbiota contributing to T2DM may include modulation of inflammation, gut permeability, and glucose metabolism. Generally, T2DM is associated with increased levels of pro-inflammatory molecules. LPS is well documented to promote low-grade inflammation. Several studies have suggested that T2DM patients have increased level of LPS in peripheral circulation.^181^ LPS can bind to TLR4, triggering macrophage aggregation and activating the NF-κB signaling pathway. This interaction leads to the release of inflammatory factors, resulting in the inhibition of insulin secretion. Gut microbiota can metabolize primary bile acids into secondary bile acids. Secondary bile acids bind to the farnesoid X receptor and release fibroblast growth factor, FGF19/15, which is able to promote insulin sensitivity and glucose tolerance.^182^ Therefore, dysbiosis of gut microbiota may lead to abnormal bile acid metabolism by affecting glucose metabolism. Another class of important metabolites from gut microbiota are SCFAs. Studies suggest that SCFAs play an important role in mediating glucose metabolism and insulin sensitivity via multiple signaling pathways. For instance, SCFAs can bind to Free Fatty Acid Receptor FFAR2 or FFAR3 on intestinal L cells, stimulating the release of glucagon-like peptide-1 (GLP-1) and peptide YY, which are known to promote insulin secretion and reduce glucagon.^183^ In addition, butyrates can protect the integrity of the intestinal barrier, which may be damaged in T2DM patients due to low-grade inflammation. Moreover, SCFAs are important anti-inflammatory mediators that can limit autoimmune response by promoting the production of regulatory T cells.^184^ Therefore, the reduced abundance of SCFA-producing bacteria may contribute to the development of T2DM.

Oral microbiota may also play a role in T2DM. Oral bacteria can translocate to the gut, changing the composition of gut microbiota and potentially mediating immune response.^185^ Several studies have identified significant alterations in oral microbiota composition between T2DM patients and healthy controls. Interestingly, Xiao et al. reported that T2DM induces a shift in oral microbiota composition with enhanced IL-17 level.^186^ By transferring to GF mice, the DM-modified oral microbiota is more pathogenic, indicating that DM can increase the risk and severity of periodontal disease.

#### Gestational diabetes mellitus

In GDM, several studies reported that gut microbiota mediates insulin resistance and inflammation during pregnancy. Metabolic disorders are commonly seen in GDM women, including enhanced insulin resistance and downregulated insulin secretion.^187^ During pregnancy, the composition of gut microbiota undergoes substantial changes, which may account for the development of GDM. For example, positive correlation has been identified between insulin and *Collinsella*, gastrointestinal polypeptide and *Coprococcus*, and adipokine with *Ruminococcaceae* and *Lachnospiraceae.*^188^ Moreover, Koren et al. demonstrated that gut microbiota changed from first to third trimesters, with increased diversity and decreased richness.^189^ It was shown that GDM patients had increased *Firmicutes* to *Bacteroidetes* ratio, an important factor that facilitates obesity and aggravates inflammation.^190^ The abundance of SCFA-producing bacteria was significantly lower in GDM pregnancies compared with healthy controls, indicating that the elevated blood glucose levels may be caused by microbiota alteration.^191^ Studies also revealed that the gut microbiota composition in the offspring of GDM mothers were different from those in non-GDM mothers. Ponzo et al. reported that the abundance of proinflammatory bacteria was higher in GDM infants than in healthy controls.^192^ Other studies confirmed this finding that the GDM infants had lower α-diversity compared with the control group, and the abundance of certain lactic acid bacteria may be affected by maternal GDM status.^193^ Therefore, gut microbiota may play a critical role in the development of GDM and may also affect GDM infants.

### Respiratory diseases

Respiratory diseases are a group of diseases that affect the lungs and other parts of the respiratory system and include chronic diseases (asthma and chronic obstructive pulmonary disease (COPD), pulmonary fibrosis) and pneumonia. Extensive studies have suggested that oral, lung, and gut microbiota are associated with the development of respiratory diseases. In this section, we will discuss the major findings demonstrating a connection between the microbiota and the development of respiratory diseases.

#### Chronic respiratory diseases

COPD and asthma are the two most frequently diagnosed chronic respiratory diseases. COPD is defined as a disease state characterized by the presence of airflow limitation associated with chronic bronchitis or emphysema. Asthma is a heterogeneous syndrome of chronic airway inflammation characterized by bronchial hyper-responsiveness to environmental triggers and by symptoms including wheezing, shortness of breath, and chest tightness. Accumulating data suggest that lung microbiota is actively involved in the development of chronic respiratory diseases. The composition of lung microbiota was found to be distinct between patients and healthy individuals. Using 16s rDNA sequencing technology, studies have identified that asthma patients had higher bacterial load and diversity, increased abundance of *Proteobacteria*, and decreased abundance of *Bacteroidetes* and *Firmicutes.*^110^ In addition, Woerden et al. found a different pattern of fungi, particularly *Malassezia pachydermatis*, in the sputum samples of asthma patients and controls. However, the research on fungi is limited and remains inconclusive.^194^ Other studies identified altered abundance of *Pseudomonas*, *Moraxella*, *Lactobacillus*, and *Haemophilus* during COPD exacerbations.^195^ Recent studies also found a potential relationship between pulmonary fibrosis and viral and bacterial infection. A clinical trial reported that the progression of pulmonary fibrosis is associated with specific *Staphylococcus* and *Streptococcus* bacterial species.^196^ Chronic inflammation induced by lung microbiota may be the key process in the initiation of chronic respiratory diseases. In asthma patients, *Proteobacteria* has been associated with hyper-responsiveness and Th17/IL-17-mediated inflammation.^197^ *H. influenza*, the most isolated pathogen from asthma patients, can induce steroid-resistant neutrophilic allergic airway diseases.^198^ Alnahas et al. demonstrated that *Proteobacterium M catarrhalis* can exaggerate allergic airway diseases by triggering a strong immune response characterized by neutrophilic infiltration, high levels of IL-6 and TNF-α, and moderate levels of Interferon (IFN)-γ and IL-17 in a mouse model.^199^ Furthermore, Garcia-Nuñez et al. reported that the bronchial microbe *Proteobacteria* may induce chronic inflammation and predict high disease severity.^200^ These studies highlighted that lung microbiota dysbiosis may potentially be associated with the development of chronic respiratory diseases.

Gut microbiota is a potent modulator of pro-inflammatory and autoimmune responses, leading to different inflammation-related diseases. Multiple studies have linked the dysbiosis of gut microbiota early in life to increased risk of asthma later in life, known as the gut-lung axis. The gut-lung axis in chronic respiratory diseases has been extensively studied and reviewed.^112^ It is suggested that gut microbiota dysbiosis in early life may lead to the development of respiratory diseases, since gut microbiota plays an important role in immune cell maturation and pathogen resistance.^201^ Indeed, Roussos et al. and Rutten et al. demonstrated that patients with chronic GI diseases have higher prevalence of chronic respiratory diseases including asthma and COPD, while the mechanisms are still unclear.^202,203^ Sprooten et al. reported that patients with acute COPD exacerbations had increased GI permeability, suggesting that gut microbiota is involved in exacerbations.^204^ Another study demonstrated that the increased levels of gut microbiota-dependent circulating TMAO were associated with all-cause mortality in COPD patients.^205^ Arrieta et al. discovered that the abundance of *Veillonella*, *Faecalibacterium*, and *Lachnospira* were significantly decreased in children at risk of asthma.^206^ It is suggested that the gut bacterial metabolites may contribute to asthma through its immune modulation. For example, Roduit et al. reported that children with high level of SCFAs are less likely to have asthma at later stage.^207^ SCFAs have been shown to promote peripheral regulatory T-cell generation^208^ and ameliorate inflammation in allergic asthma models.^209^ In addition to bacterial metabolites, it is suggested that lymphocytes with altered homing properties may contribute to asthma.^210^ Under normal situation, lymphocytes are thought to exhibit tissue specificity to the site where they first encounter the antigen. However, intestinal lymphocytes from IBD patients are known to lack tissue specificity and may account for the presence of inflammation in organs other than the gut. Huang et al. reported that innate lymphocytes were recruited from the gut to the lungs following inflammatory signals from IL-25.^211^ Interestingly, some data indicates that the gut-lung axis may have a bidirectional interaction. Perrone et al. showed that pneumonia induced intestinal epithelial apoptosis^212^ and decreased intestinal epithelial proliferation^213^ in mice.

Oral microbiota has been associated with chronic respiratory diseases due to the contiguous anatomic structure and microaspiration.^214^ Early studies found significant similarity between the oral and lung microbiota, while the nasal microbiota shares less similarities with lung microbiota.^215^ It is hypothesized that the oral microbiota may contribute to chronic respiratory diseases through aspiration and systemic inflammation. It is possible that aspiration of oral bacteria into the lung leads to lung microbiota dysbiosis and inflammation. Segal et al. reported that the enrichment of oral bacteria *Veillonella* and *Prevotella* in bronchoalveolar lavage samples has been associated with subclinical inflammation, characterized by increased neutrophils and lymphocytes.^216^ A RCT showed that bronchial microbiome of asthmatic subjects was uniquely enriched with two periodontal pathogens, *Fusobacterium* and *Porphyromonas.*^217^ Many periodontitis-related inflammatory cytokines have also been detected in chronic respiratory diseases. Aaron et al. reported that TNF-α was increased in the sputum of COPD patients.^218^ Substantial studies have shown that TNF-α can stimulate the generation of ROS in pulmonary tissues, accompanied by the generation of various adhesive and proinflammatory molecules such as VCAM-1, ICAM-1 and RAGE. TNF-α is also suggested to function as a pro-inflammatory cytokine in asthma that recruits neutrophils and eosinophils.^219^ Periodontitis is related to high levels of systemic inflammatory markers, such as CRP and IL-6. Jousilahti et al. reported that the level of CRP was significantly associated with asthma prevalence.^220^ However, the oral-lung axis has not been fully understood and deserves further investigation.

#### Pneumonia

The normal respiratory tract and gut microbiota protect against pneumonia by preventing pathogenic bacteria colonization and by modulating immune responses. Therefore, it is not surprising that the dysbiosis of respiratory tract microbiota is considered a risk factor of pneumonia.

The upper airways are the main source of microbes to the lower airways. Recently, researchers have shown that the reduction of nasal microbiota diversity increased susceptibility to pneumonia. Particularly, three microbiota profiles dominated by *Lactobacilli*, *Rothia*, and *Streptococcus* were significantly associated with pneumonia.^221^ In neonates, the pathogenic bacterial colonization of the airways with *S. pneumonia*, *H. influenza*, and *M. catarrhalis* were associated with increased risk of pneumonia and bronchiolitis.^222^ Regarding the lower airway microbiota, studies suggested that increased abundance of *Prevotella* and *Veillonella* predisposed pneumonia in HIV patients.^223^ In addition, altered immune response due to microbiota dysbiosis may increase the risk of pneumonia. For example, dysregulation of SCFA-producing bacteria may contribute to the development of pneumonia. Segal et al. suggested that pulmonary SCFAs correlated with increased anaerobic bacteria.^224^ Indeed, SCFAs have a direct inhibitory effect on immune response via suppression of IFN-γ and IL-17A pathways. During bacterial infection, neutrophils are rapidly migrated to lung parenchyma and alveolar. The IFN-γ released by neutrophils regulates bacterial clearance, therefore the level of IFN-γ is critical for host defense during pneumonia.^225^ Similarly, Th17 cells and its signature IL-17A signaling is an important immune response against pneumonia. During infection, IL-17A acts on nonimmune cells to trigger the release of antimicrobial proteins, cytokines, and chemokines, thus enhance innate immunity during microbial infection.^226^ By inhibiting the IFN-γ and IL-17A pathways, it allows the lung bacteria reproduction and worsen inflammation. Salk et al. reported that the influenza-specific lgA production is significantly associated with levels of *Lactobacillus*, *Prevotella*, *Veillonella*, *Bacteroide*, and *Streptococcus.*^227^ Interestingly, recent studies suggested that commensal microbes can play a crucial role in the development of pneumonia. Recently, the global pandemic COVID-19 has become a major research area in respiratory disease. Emerging data are now connecting the COVID-19 mortality with microbiota dysbiosis. Fan et al. investigated the lung microbiota characteristics from 20 deceased COVID-19 patients.^228^ It is suggested that the dysbiosis of lung microbiota is characterized by increased abundance of *Acinetobacter* spp., which are related to multidrug resistance and mortality. In addition, *Cryptococcus* was the dominant fungi in the lung fungal communities, along with *Issatchenkia*, *Cladosporium*, *Candida*, etc. Han et al. reported that COVID-19 may induce severe dysbiosis of lung microbiota, particularly with increased abundance of *Klebsiella oxytoca*, *Faecalibacterium prausnitzii*, and *Rothia mucilaginosa.*^229^ Segal et al. revealed that the enrichment of lower airways with oral bacteria *Mycoplasma salivarium* was associated with poor clinical outcome.^230^ However, no significant connection was found between increased mortality and secondary respiratory pathogens.

Gut microbiota is another major subject when studying pneumonia-microbiota interaction. Schuijt et al. identified that the gut microbiota plays a protective role against *S. pneumoniae* infection.^231^ Compared with the control group, *S. pneumoniae* infection in gut microbiota depleted C57BL/6 mice demonstrated increased bacterial dissemination, inflammation, organ damage and mortality. In addition, depletion of gut microbiota was associated with the upregulation of metabolic pathways, leading to reduced responsiveness to inflammatory cytokines. In accordance, Felix et al. showed that the commensal gut segmented filamentous bacteria protected immunodeficiency mice from *S. pneumoniae* infection. It is likely that the bacteria promoted a shift in lung neutrophil phenotype from inflammatory to pro-resolution, which is similar to heat-inactivated *S. pneumoniae* treatment. Recent data also suggested that gut microbiota composition may reflect disease severity in COVID-19 patients.^232^ In COVID-19 patients, decreased abundance of several gut commensals was observed, including *Bifidobacteria*, *Eubacterium rectale*, and *Faecalibacterium prausnitzii*. The dysbiosis gut microbiota was positively associated with disease severity, with elevated levels of inflammatory cytokines and blood markers such as CRP, aspartate aminotransferase, and lactate dehydrogenase. Therefore, unlike in chronic respiratory diseases, the gut-lung axis may provide additional protection for the host against pneumonia by regulating the immune response.

### Inflammatory bowel disease

IBD is a chronic and remittent inflammatory condition of the GI tract, encompassing several diagnoses including Crohn’s disease (CD) and ulcerative colitis (UC).^233^ While UC is known as continuous, diffuse, and superficial inflammation of the colon, CD is characterized by discontinuous, transmural lesions affecting different regions of the GI tract.^234^

Although the development of IBD is due to complex multifactorial mechanisms, several risk factors have been extensively studied and are now well documented. The pathogenesis of IBD involves dysregulated immune response, genetic mutations, and environmental factors.^235^ The intestinal barrier plays an important role in maintaining homeostasis; dysfunction of the barrier may lead to ulceration. Specifically, the intestinal barrier would be susceptible to pathogen invasion without the secretion of antimicrobial peptides (AMPs) or tight junction proteins.^236^ During initial disease in genetically susceptible individuals, the immune response is altered, leading to loss of immune tolerance to intestinal antigens. This subsequently stimulates the differentiation of helper T cells and release of chemokines and proinflammatory cytokines, which induce chronic inflammation of the intestine.^237^ In addition to immune dysregulation, genetic factors are involved in determining IBD development. For example, genetic mutations associated with CD include polymorphisms for the Nucleotide Oligomerization Domain Containing 2 (NOD2/CARD15), Immunity-elated GTPase family M (IRGM), and autophagy-related 16 Like 1 (ATG16L1).^238^

Studies have shown that gut microbiota are highly associated with the development of IBD. Mechanistically, microbiota dysbiosis is linked to IBD through its impact on inflammation as well as the intestinal barrier. As described before, microbiota dysbiosis can induce chronic inflammation, which is associated with the development of multiple diseases such as cancer, diabetes, and heart diseases. Importantly, it is postulated that microbiota can interact with intestinal barrier and lead to IBD. For example, Kleessen et al. reported that bacterial invasion of the mucosa was detected more in IBD patients than in controls.^239^ It was also reported that the abundance of adherent-invasive *E. coli* was significantly increased in CD patients, suggesting that the pathogenic bacteria may affect the permeability of the intestine, the composition of gut microbiota, and eventually induce intestinal inflammation.^240^ In healthy individuals, the predominant phyla are *Firmicutes* and *Bacteroidetes*, followed by *Proteobacteria* and *Actinobacteria*. Multiple studies have revealed that the composition of gut microbiota is different between IBD patients and healthy controls.^241^ For example, the ratio of *Bacteriodetes* to *Firmicutes* is decreased while the abundance of gammaproteobacterial increased in IBD patients.^242^ The protective and normal bacteria, *Bacteroides*, *Eubacterium*, and *Lactobacillus* are significantly reduced in CD and UC patients.^243^ A meta-analysis study suggested that enterohepatic *Helicobacter* species, but not intestinal H. pylori infection, was significantly related to IBD.^244^ It should be noted that, although many studies provided the association between microbiota dysbiosis and IBD, the causation remains to be determined.^245^ It is possible that the microbiota dysbiosis can be considered a response to the environmental changes due to intestinal inflammation. The possible role of fungi and viruses in IBD are also being studied and reviewed, but no link has been established thus far.^234,245^

While most of the studies are focusing on gut microbiota, oral microbiota is gaining attention with the characterization of the oral-gut axis. Kitamoto et al. showed that pathobionts and pathogenic T cells of oral origin were able to translocate and colonize in intestines, causing IBD in periodontitis mouse models.^246^ Derrien et al. concluded that the bacteria residing in the oral cavity and GI tract maintain intimate relationships,^247^ supporting the notion of an oral-gut axis. Recently, a meta-analysis by She et al. demonstrated that periodontitis was significantly associated with IBD, while the mechanisms are undetermined.^248^ Another study by Kimura et al. suggested that, in the salivary microbiota of IBD patients, the abundance of *Bacteroidetes* was significantly increased with a concurrent decrease of *Proteobacteria.*^249^ They also found a significant correlation between inflammatory cytokine levels and the abundance of *Streptococcus*, *Prevotella*, *Veillonella*, and *Haemophilus*, implicating a possible relationship between dysbiosis of oral microbiota with inflammatory response in IBD patients. A case-control study by Vavricka et al. showed that both periodontitis and gingivitis marker levels were increased in CD patients compared with healthy controls.^250^ Although these studies have established an association between oral diseases with IBD, data regarding oral microbiota in IBD is still limited and require further investigation.

### Brain disorders

Neuropsychiatric and neurodegenerative disorders of the brain, along with many other comorbidities, were known to be responsible in causing significant mortality in different population subsets. Extensive research over the years have shown to implicate the role of microbial diversity in brain disorders by modulating the factors linked with the development of these disorders. One example is data from a meta-analysis study which shows that depression is responsible for an increase in relative risk of mortality from all causes, specifically about 1.86 times more than non-depressed patients.^251^ Microbiota-induced hyperactivity of the HPA axis and inflammation are also shown to be associated with provoking depression.^252^

#### Neuropsychiatric disorders

Gut microbiota is believed to play a vital role in mediating neuronal behavior via gut-brain axis.^253^ Preclinical studies have established that gut microbiota affects cognitive performance, repetitive behaviors, and social interactions in different animal models.^62,254,255^ One of the plausible hypotheses about gut microbiota’s involvement in affecting neuronal disorders is described by stress-induced intestinal permeability, permitting endotoxins to enter the blood circulation, thereby triggering an immune response.^256^ This peripheral inflammation can also influence mental health by promoting the entry of neurotoxins into the brain and also by obstructing neurotransmitter systems.^257^ Although the direct mechanism of gut bacteria influencing neuropsychiatric disorders was clearly not studied, many studies believe that gut-induced stress has a vital role along with disrupted gut microbiome and various other factors in causing depression, anxiety, and other psychological disorders. Recently, Jiang et al. has demonstrated that fecal samples from patients with major depressive disorder have shown increased *Bacteroidetes, Protobacteria* and *Actinobacteria* along with less *Firmicutes* when compared with fecal samples from healthy controls.^258^ Decreased expression of certain families such as *Lachnospiraceae* and *Ruminococcaceae* within the phylum *Firmicutes* was reported, and this is believed to be correlated with behavioral changes caused by stress. Some bacterial genera such as *Roseburia*, *Blautia*, *Lachnospiraceae*, and *Ruminococcaceae* are associated with synthesis of SCFA (responsible for barrier integrity) and anti-inflammatory properties. It was believed that there was an extensive correlation between diversity of gut microbiota and mood-related behaviors, especially depressive disorder.^259^

Both major brain disorders, depression and anxiety are indicated to be influenced by stress-regulated HPA axis pathway, which is believed to be strongly modulated by gut microbiota composition.^257,260^ An epigenetic study using GF mouse models had demonstrated that GF mice showed significant difference in gene expression in the brain systems when compared with control mice, notably in the areas of cortex, cerebellum, striatum, and hippocampus.^261^ It is suggested that the gut-brain axis may be affected by regulation of stress hormones and the establishment of neuronal circuits. Based on this initial finding, many studies have investigated the substantial changes observed in GF mice compared with the wild-type controls. In GF rats, increased levels of neurotransmitters such as norepinephrine, dopamine, and serotonin were reported in the striatum, whereas dopaminergic turnover was found to be decreased in the frontal cortex, striatum, and hippocampus of GF rats.^262^ Few other study findings using GF mice models have demonstrated a reduction in brain-derived neurotropic factor and nerve growth factor-inducible protein A in several brain regions, and an increase in synaptophysin and post synaptic density (PSD-95) proteins in GF mice brain systems when compared with controls.^261^ When it comes to autism spectrum disorder (ASD), several pre-clinical studies have reported that gut dysbiosis induced significant neurodevelopmental changes in mouse models of ASD.^263^ Species like *Clostridium* and *Ruminococcus* was found to be different when compared between autism children and controls.^264^ Adams et al. demonstrated that symptoms of GI discomfort were correlated with severity of autism in children.^265^ A small pilot scale study conducted by Kang et al. using fecal transplantation of standardized gut microbiota to children diagnosed with autism spectrum disorder has enhanced GI function and decreased behavioral ASD scoring.^266^

#### Neurodegenerative disorders

Lately, accumulating body of evidence from various studies are emphasizing the importance of gut microbiota in the progression of various neurological disorders such as Alzheimer’s disease (AD), cerebrovascular stroke (CVS), Parkinson’s disease (PD), and schizophrenia etc. It has been evident that gut microbes are involved with regulation of brain function via its effect on host innate immunity.^267^ The community composition is also found to be varied depending on the way of newborn delivery. Vaginally delivered newborns are colonized with microbiota from the maternal genital tract and is more heterogenous compared with newborns delivered by Cesarean section.^268^ Cesarean delivered newborns displayed less brain electrical activity, which is supported by in vivo studies using Cesarean-delivered rats, where these rats exhibited pre-pubertal alterations in the development of cortex and hippocampus.^269^

During the presymptomatic stages of PD, α-synuclein-mediated Lewy body pathology was observed in the ENS and dorsal motor nucleus of the vagus nerve. A total of 38 human fecal samples were analyzed using 16s rRNA sequencing, displaying significant differences in bacterial composition such as decreased *Blautia*, *Faecalibacterium* and *Ruminococcus* and increased *Escherichia-Shigella*, *Streptococcus*, *Proteus*, and *Enterococcus.*^270^ Li et al. in a study conducted in China, has identified significant diversity in different taxa such as increases in *Prevotella*, *Akkermansia* and decreased abundance in *Lactobacillus species* in PD patients when compared with healthy controls.^271^ These species play a prominent role in affecting the harmony of gut homeostasis, for example, increased numbers of *Akkermansia* were shown to be responsible for increased intestinal permeability and facilitating pathogen entry.^272^ Increased level of certain *Prevotella* species is associated with mucin synthesis in the gut mucosal layer and production of SCFAs, which are shown to mediate neuroinflammation in mouse models of PD.^67^ However, another study has reported a decreased abundance of *Prevotella* in PD patients compared with healthy controls, which led to the need of additional studies or bigger sample size to understand the specific role of *Prevotella* and its family in progression of PD.^273^ Differences in genotype, diet, and lifestyles of the population subsets might be a potential reason for this disparity in reports. Pathological features of AD were characterized by the presence of amyloid-β plaques and intracellular tau based neurofibrillary tangles (NFT). Vogt et al. have reported significantly less microbiome diversity in AD patients compared with healthy controls. Particularly in this study, a decrease in phylum like *Firmicutes, Actinobacteria* (member of *Bifidobacterium*) and an increase in phylum of *Bacteroidetes* and *Proteobacteria* were observed in the AD group.^274^ Reduction in *Firmicutes* and *Bifidobacterium* has been well studied for their association with T2DM and inflammation, which are identified as major risk factors for AD.^179^ Inflammatory intestinal bacterial taxa are found to be associated with high level of inflammatory cytokines, including IL-6, TNF-α, CXCL2, NLRP3, and brain amyloidosis in a study conducted in older people suffering from cognitive disorders.^275^ In the same study, increased levels of pro-inflammatory cytokines were observed with increased numbers of *Escherichia/Shigella*. Colonization of certain pathogenic bacterial strains such as *Toxoplasma* and *Chlamydiaceae pneumoniae* has also been suggested for their roles in chronic neuroinflammation and NFT in AD.^276^

CVS is a major neurological condition associated with neurological defects and impairment in cognitive functions leading to disability and mortality. Acute middle cerebral artery occlusion-induced stroke mouse models have shown reduced species diversity and increased growth of *Bacteroidetes* in mice, while fecal transplantation of normal gut microbiota normalized brain lesion-induced dysbiosis and improved stroke outcomes.^277^ Another preclinical study using mice has reported a significant change in cecal microbiota, such as *Prevotellaceae* and *Peptococcaceae*, the former of which is a core part of microbiota in mice, although their functionality in humans is yet to be identified.^278^ Additionally, experiencing stress before stroke might increase the bacterial translocation from the intestine to the blood stream, triggering immune responses.^279^ Despite the number of studies done in strengthening the idea of gut microbiota involvement in orchestrating neuronal harmony, additional studies are required to identify the clinical benefits of targeting specific microbiota in treating these conditions.

#### Oral and respiratory microbiota in brain disorders

Oral microbiome is another key contributor to the development of neurological disorders, as improper maintenance of oral health can influence the growth of complex communities on the surface of teeth, tongue, or under the gum.^280^ Hicks et al. has reported that salivary microbiome analysis has shown wide differences between ASD, typically developing, and non-developmentally delayed groups of children.^281^ In a depression-related study, 1S rRNA gene-based next-generation sequencing was used to profile the bacterial composition of saliva in depressed patients compared with young adults; it has shown diversification but importantly, increased *Prevotella nigrescens* and *Neisseria* was observed in depressed individuals.^282^ Smoking and alcohol consumption are two major factors that induce dysbiosis in oral microbiota and promote growth of pathogenic bacteria.^280^ A meta-analysis study has revealed that drinking alcohol is associated with pathogenesis of AD and also with significantly decreased level in *Firmicutes* phyla and an increased level in *Bacteroides* phyla in these patients.^283^ In a cross-sectional case control design study on PD, around 16 bacterial families were found to be altered in early-stage PD patients.^284^ Among them, variation in families like *Bifidobacteriaceae, Saccharomycetaceae* and *Lactobacillaceae* were studied extensively for their role in progression of PD. A cohort study with 68 patients comprising of AD and control groups have reported differential abundance of two specific taxa *Pasteurellaceae* and *Lautropia mirabilia*, which were found to be associated with mild cognitive impairment.^285^ Additionally, another study conducted in 78 patients have revealed increased relative abundance of *Moraxella, Leptotrichia* and *Sphaerochaeta* and decreased *Rothia* in saliva of AD patients when compared with healthy controls.^286^ Unfortunately, not many studies have been reported about respiratory microbiota for its role in neurological disorders, and the research concerning respiratory microbiota is still at infancy stage. Although the affinity of microbial dysbiosis in many neurological disorders is being extensively studied, currently there is no gold standard to interlink the changes in microbial environment with the pathogenesis of these disorders. More preclinical and clinical studies targeting microbiome are required to understand the extent and complex nature of microbiome’s association with the development of several brain disorders.

### Chronic kidney diseases

Around 9% of the global population suffer from chronic kidney disease (CDK).^287^ Co-morbidities like diabetes, hypertension and heart disease are considered some of the major risk factors for CKD.^288^ CKD is physiologically identified as a decrease in glomerular filtration rate (GFR) < 60 ml/min per 1.73 m^2^ or by the existence of albuminuria for 3 or more months. Gradual loss of kidney function and irreversible renal structural changes are the main characteristics observed in CKD patients.

#### Gut-kidney axis communication and gut microbiota

Differences in microbial ecosystems were studied persistently for their involvement in the progression of CKD.^289^ Recently, oral microbiota were extensively studied for the role in mediating chronic systemic inflammatory dysregulation. It has been reported that conditions affecting oral microbiota like periodontitis indirectly affects CKD by augmenting systemic inflammation.^290^ Biomarker-based human studies have reported that elevated IgG levels due to the presence of elevated periodontal pathogen species like *P. gingivalis*, *T. denticola*, *S. noxia*, *A. actinomycetemcomitans*, and *V. parvula* are connected to detrimental kidney function.^291^ Bastos et al. has reported that higher frequency of *Candida albicans, P. gingivalis, T. forsythia*, and *T. denticola* was associated with the development of chronic periodontitis in CKD patients, thereby indicating a bidirectional relationship between changes in oral microbiota and CKD.^292^ A large 10-year cohort study with CKD patients suffering with periodontitis had demonstrated an increase in mortality rate from 32% to 41% in those patients.^293^ However, data is lacking to establish a solid confirmation on the role of oral microbiota in the pathogenesis of CKD.

The gut-kidney axis functionality is based on metabolic and immune pathways being interlinked with each other.^288^ The metabolic pathway is mostly focused on gut microbiota-produced metabolites that mediate host physiological functions, whereas the immune pathway depends on several other components like monocytes, lymphocytes, and cytokines, which facilitate the communication between the gut and kidney.^294^ Recently, involvement of dysbiosis in the gut microbial environment leading to CKD has garnered attention, as there are implications of cross functionality between the gut and renal system.^295^ Numerous studies have been conducted to link the qualitative and quantitative changes in intestinal microbiota with the pathogenesis of CKD and end-stage renal disease (ESRD).^296,297^ However, there is no solid evidence confirming the presence of altered gut microbiota in CKD patients.^297^ Factors such as increased protein absorption, reduced dietary fiber intake, slower intestinal transit, and frequent oral intake of iron supplements and antibiotics resulted in altered intestinal microbial environment, leading to systemic inflammation and accumulation of uremic toxins. Both inflammation and uremic toxins substantially contribute to the progression of CKD and CKD-associated complications.^298^ Vaziri et al. showed that continuous loss of kidney function augments intestinal dysbiosis in CKD and ESRD patients.^299^ A comparative study between fecal samples comparing healthy subjects with CKD patients have exhibited that CKD patients show reduced abundance of *Actinobacteria* phylum and *Akkermansia* genera, where the latter is correlated with regulating levels of IL-10, denoting its importance in systemic inflammation.^300^ Another clinical study conducted using 73 subjects have identified 31 phylotypic differences between CKD and control groups with phylotypes like *Bacteroides, Parabacteroides, R. gnavus, R. torques, Flavonifractor, Weissella, Ruminiclostridium, Erysipelatoclostridium, Eggerthella*, and *Sellimonas* being predominant in CKD patients.^301^ ESRD patients have shown an increase in *Actinobacteria, Proteobacteria* and *Firmicutes* and a decrease in *Bifidobacteria* and *Lactobacilli* compared with the control group.^299^ Another study has demonstrated that changes in gut microbiota is also shown to be an important factor in contributing to inflammation along with oxidative stress by increasing accumulation of gut-derived uremic toxins such as indoxyl sulfate, amines, ammonia, p-cresyl glucuronide (PCG), p-cresyl sulfate (PCS) and TMAO in CKD patients.^302^ Dietary intervention is also an additional variable that induced post-translational modification of uremic toxins, indirectly contributing to CKD progression.^303^ Fiber-rich diet is a main contributor to colonic bacterial fermentation, and CKD patients often have a low fiber intake diet to limit the potassium intake.^304^ A meta-analysis study has reported that one-third of CKD patients exhibit higher levels of pathogenic bacteria like *E. coli* and *Enterobacter*, and mild CKD patients have shown increasing presence of uremic toxin-producing bacteria.^305^ In vivo studies using collagen type 4α3 (*Col4a3*)–deficient mice demonstrated that uremia is associated with intestinal dysbiosis and intestinal barrier dysfunction, causing persistent systemic inflammation in CKD.^306^ Human studies conducted with CKD patients have shown higher levels of PCS and PCG in general with PCS reaching levels around 200-fold higher than PCG.^307,308^ Mutsaers et al. have demonstrated that PCS and PCG affect renal tubular function while simultaneously affecting the activity of MRP4 (PCS and PCG) and BCRP (PCG) transporters.^309^ In vivo studies have shown that PCS-administered rats at a dose of 50 mg/kg for 4 weeks induced renal tubular cell damage.^310^ Various in vitro studies have also shown that indoxyl sulfate is responsible for inducing inflammatory and profibrotic responses in tubular cells.^311,312^ Increased levels of TMAO are associated with increased risk prediction of CVD, systemic inflammation, and mortality in CKD patients.^313^ A trial study using samples obtained from CKD patients displayed higher plasma levels of TMAO in CKD vs non-CKD patients.^314^ Persistent low-grade inflammation is augmented due to translocation of bacteria and bacterial products from the gut lumen to blood via increase in intestinal permeability. Decreased levels of certain microbiota metabolites like butyrate and vitamin K, which are nephroprotective, were also observed.^308^ These studies show strong evidence for involvement of various disturbances in gut-renal system communication via dysbiosis in microbiota in the progression and pathogenesis of kidney diseases.

### Chronic liver diseases

Liver diseases remain one of the leading causes of morbidity and mortality worldwide. Nonalcoholic fatty liver disease/nonalcoholic steatohepatitis (NAFLD/NASH) and alcoholic liver disease (ALD) are the most common chronic liver diseases that often lead to liver cirrhosis and cancer.^315^ NAFLD comprises a wide span of liver damages from benign steatosis to steatohepatitis with hepatocellular inflammation and damage.^315^ ALD may take the form of chronic disease state (steatosis, steatohepatitis, fibrosis, or cirrhosis) or acute involvement (alcoholic hepatitis).^316^ Cirrhosis is the end stage of all chronic liver diseases, characterized by tissue fibrosis and the transformation of normal liver architecture to abnormal nodules. Recent studies have suggested the roles that oral and gut microbiota play in the pathogenesis of chronic liver diseases.

#### Gut microbiota in liver diseases

Mounting evidence supports the bidirectional gut-liver axis, due to the fact that liver secretes bile acids into the biliary tract and receives blood supply via the portal vein.^317^ Therefore, gut microbiota may contribute to liver diseases by delivering pathogens or metabolites into the liver through the portal vein. Currently, clinical data demonstrating the relationship between gut microbiota dysbiosis and liver diseases are still limited. Mouzaki et al. reported that patients with NASH have lower level of Bacteroidetes compared with healthy controls.^318^ Raman et al. suggested a compositional shift in the gut microbiota of obese NAFLD patients. Analysis of fecal microbiome showed an increased abundance of *Lactobacillus* and selected members of *Firmicutes.*^319^ Wong et al. reported an increased fecal abundance of *Parabacteroides* and *Allisonella* but decreased levels of *Faecalibacterium* and *Anaerosporobacter.*^320^ In liver cirrhosis patients, Chen et al. showed that abundance of Bacteroidetes was significantly reduced, while Proteobacteria and Fusobacteria were enriched compared with healthy controls.^321^ However, the investigation of gut microbiota with liver diseases is mainly conducted in preclinical studies. More evidence is required to conclude whether dysbiosis contributes to liver diseases or is a consequence of the disease state.

Researchers have postulated several mechanisms linking gut microbiota to liver diseases, including regulation of bile acid metabolism, intestinal permeability, chronic inflammation, and immune response. The gut microbiota plays an essential role in the metabolism of bile acids by converting primary bile acids into secondary bile acids. Deoxycholic acid, a major secondary bile acid, has been suggested to activate NF-κB stress response pathway by generating ROS.^322^ In addition, recent studies have established the crosstalk between ROS and NF-κB signaling pathway. While high ROS level usually results in cell damage, NF-κB pathway is known to promote cell proliferation.^323^ Therefore, it is likely that deoxycholic acid can induce cytotoxicity by promoting the generation of ROS, and simultaneously activates NF-κB pathway to allow damaged cells to resist apoptosis. Hence, microbiota dysbiosis may affect to bile acid homeostasis, leading to pathogenesis of chronic liver diseases such as NAFLD/NASH. Moreover, gut microbiota is involved in the metabolism of choline, and its deficiency usually leads to hepatic steatosis.^324^ There are multiple mechanisms established to explain choline deficiency and liver diseases, including 1) accumulation of DNA damage during choline depletion, 2) overproduction of free radicals in choline deficient hepatocytes, and 3) induction of inflammatory response due to death of hepatocytes.^324^ Spencer et al. showed that the abundance of *Gammaproteobacteria* and *Erysipelotrichi* were significantly associated with choline deficiency-induced fatty liver.^325^ Impaired intestinal permeability allows the translocation of gut bacteria and their component, which is associated with chronic liver diseases. For example, the Gram-negative bacteria structural element LPS was suggested to be elevated in portal vein in ALD and cirrhosis.^326^ Mechanistically, Seki et al. described that, upon LPS binding, TLR4 upregulates chemokine secretion and downregulates TGF-β pseudoreceptor Bambi to enhance TGF-β signaling pathway.^327^ The study also suggested that the effect of LPS is mediated by MyD88-NF-κB-dependent pathway, as MyD88-deficient mice had decreased hepatic fibrosis. Gut microbiota-mediated chronic inflammation and immune activation is central to the pathogenesis of multiple diseases. Recent studies also suggested such mechanisms in the development of NAFLD/NASH.^328^ In conclusion, current research highlights the potential role of gut microbiota in liver diseases, but further study is needed to confirm their relationship.

#### Oral microbiota in liver diseases

As mentioned in other diseases, it has been demonstrated that oral microbes or their metabolites are able to invade other sites of the body. Although the correlation between periodontal and liver diseases is yet to be established, recent studies implicated that periodontal bacteria may be involved in the progression of NAFLD, NASH, and cirrhosis.^329^ Yoneda et al. reported that *P. gingivalis* (one of the most common periodontal pathogens) may influence the pathogenesis of NAFLD/NASH in a mouse model.^330^ In addition, they found that *P. gingivalis* infection was mostly observed in NAFLD patients compared with control subjects. Clinical data also support the notion that periodontitis may serve as a risk factor in the progression of NAFLD/NASH.^331^

Mechanistically, this process may be attributed to many factors including pro-inflammatory mediators, oxidative stress, and pathogen invasion. The migration of periodontal bacteria as well as their metabolites (LPS, peptidoglycans, etc.) into the systemic circulation is usually recognized by TLRs, which leads to the activation of T cells and the release of pro-inflammatory cytokines, chemokines, and ROS/RNS.^332^ This may indicate the oral-gut-liver axis in the inflammation pathway as suggested by Acharya et al.^333^ They proposed that the gut with impaired intestinal permeability may act as an intermediate between oral microbiota and the liver. Therefore, after the bacteria and metabolites enter the systemic circulation, they can reach the liver and bind to innate TLRs of hepatocytes and Kupffer cells, inducing inflammation and causing liver diseases.^334^ In addition, Silva Santos et al. observed that cirrhotic patients exhibited numerous oral diseases other than periodontitis, such as candidiasis, xerostomia, and petechiae.^335^ Moreover, dysbiosis of oral microbiota has been suggested to promote the pathogenesis of hepatitis B virus (HBV)-induced chronic liver disease. HBV-associated oral bacteria including *Fusobacterium*, *Eubacterium*, and *Treponema* may invade and contribute to the dysbiosis of gut microbiota as opportunistic pathogens, which subsequently participate in the formation of liver diseases.^336^

## Microbiota and disease treatment

**Fig. 5:**
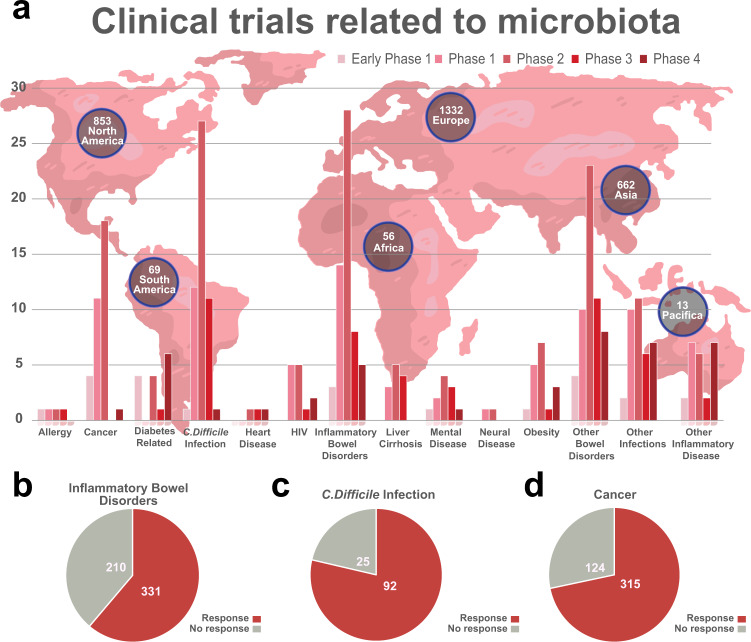
Current number of microbiota-related clinical trials by regions and study phases. Data are updated until October 2021

**Fig. 6:**
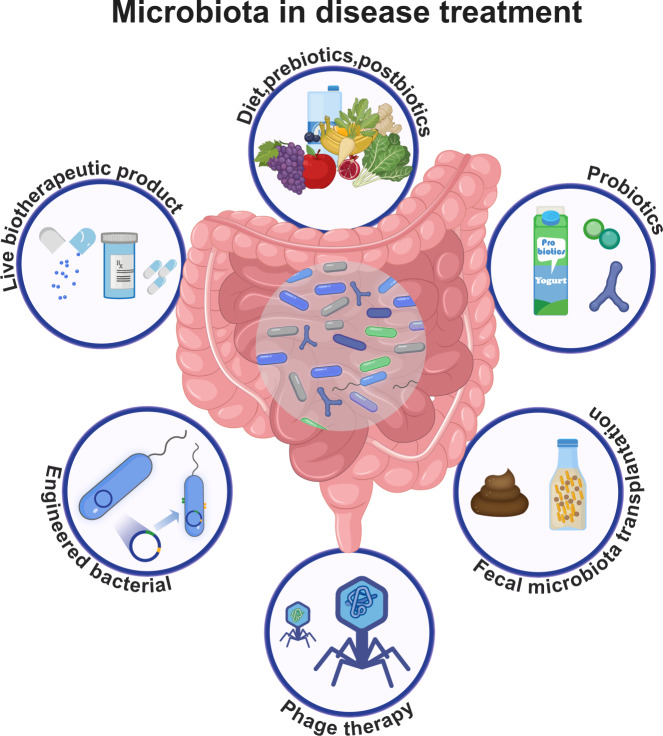
Strategies to modify gut microbiota for disease treatment

With the gradual understanding of microbiota, the potential of treating diseases through manipulating microbiota has attracted people’s attention. Because the human gut is involved in a wide range of physiologic functions, its modulation is expected to prevent or treat the corresponding diseases. Therefore, climbing number of clinical trials are ongoing to investigate this possibility (Fig. 5a). The majority of clinical trials focusing on efficacy of fecal microbiota transplantation (FMT) is various diseases. Since *C.Difficile* infection, cancer, and IBD has the highest number of trials, we also summarized the data from pubmed (Supplemental Table S1). As shown in Fig. 5b, c, FMT treatment in IBD and *C.Difficile* infection showed a significant response rate compared to placebo treatment. Similarly, probiotics treatment as an adjuvant therapy in cancer patients also demonstrated an optimal result (Fig. 5d). It should be noted that while the response rate is promising, the trials are mainly pilot studies with small sample size. In addition, the underlying mechanism for complete response required further investigation to optimize the experimental design and to personalize the treatment. Diet is considered the main short-term and long-term regulator of the gut microbiota,^337^ along with healthy lifestyle habits. As shown in Fig. 6, this section will present the latest clinical interventions targeting the gut microbiota, including microbiota modulations, FMT, and bacteria engineering. We will also discuss the pharmacological microbiota–drug interactions in clinical settings.

### Microbiota modulations

#### Probiotics and prebiotics

Generally, probiotics and prebiotics are the most popular topic in microbiota modulation research. They are often used as a dietary supplement for clinical intervention by oral administration. Differences in dosage form and host are considered to be the main factors affecting the effectiveness of probiotics and prebiotics.^338^ Probiotic administration is suggested to restore microbial dysbiosis and maintain intestinal microbial balance by occupying host tissue and preventing colonization of pathogenic bacteria. Extensive research has indicated the potential mechanism of probiotics in disease treatments, including differences in various probiotic strains and mucosal immune system, regulation of host metabolism or altering intestinal neuromuscular function.^339^ However, clinical data is insufficient to support their role. Several clinical trials denied the benefits of probiotics in cancer treatment.^340^ Importantly, patients with damaged intestinal barrier and/or compromised immune systems might have a probiotic translocation. Some published case reports associate negative effects of probiotics with conditions such as bacteremia, fungemia, endocarditis, liver abscess and pneumonia,^341^ which compels us to ponder the actual effects of probiotics.

Prebiotic was recently redefined as “a substrate that is selectively utilized by host microorganisms conferring a health benefit” in 2017. Prebiotics were originally used to study the stimulating effect of probiotics. The most well-known prebiotics are inulin, fructo-oligosaccharides (FOS), lactulose, and galacto-oligosaccharides (GOS). Prebiotics are mainly used to modulate the strains of *Bifidobacterium* and *Lactobacillus*, which produce lactic acid and acetate, and to maintain the health of the host by fermenting prebiotics.^342^ Studies have confirmed that taking prebiotics can stimulate the selective enrichment of probiotics in the intestinal tract, thereby regulating immune response and preventing pathogens.^70^ Although some basic studies have confirmed that prebiotics can inhibit the colonization of pathogens by mimicking glycoconjugates of microvilli,^343^ and even directly act on the intestinal tract to regulate immunity,^344^ the applicability of prebiotics as a clinical intervention is still debatable. Belcheva et al. suggested that supplementation with prebiotic/butyrate could promote tumor progression due to genetic variation in individuals.^345^ Their findings suggest that butyrate functions as an oncometabolite, while a substantial of studies reported butyrate as tumor suppressive metabolite. Indeed, butyrate is known to exhibit differential effects toward normal and cancerous colonocytes. In colon cancer cells, butyrate is metabolized to a lesser extent compared to normal cells, thereby accumulating as HDAC inhibitor to inhibit cell proliferation and induce cell apoptosis.^346^ The difference observed in this study may lay between host genetic background, the age, and the presence of other bacterial metabolites. In addition, the study was performed in mice model, so the translation to human requires further investigations.

Sasaki et al. showed that transglucosidase, which generates prebiotics, can reduce high blood sugar levels in patients with T2DM and inhibit weight gain.^347^ Because probiotics and prebiotics are cheap and easy to handle, they are often used in the care of patients with AD. Long-term supplementation with milk enriched with *Bifidobacteria* and *Lactobacillus* fermentum improves learning and memory in AD patients.^348^ In CRC treatment, some probiotic strains could be beneficial as an adjuvant therapeutic agent, such as multigene and multistrain probiotics, including *B. breve, B. infantis, B. longum, L. acidophilus.*^349,350^ Recent studies in intestinal inflammatory disorders show that probiotics might have some efficacy in UC and pouchitis, but with insignificant effect in CD. Probiotic supplementation may significantly reduce rates of rotavirus diarrhea, although the curative effect of probiotics in NSAID enteropathy and IBS is controversial.^351^ This is because the study populations, types of probiotics and dosage and length of follow-up various greatly between the clinical studies. Similarly, the treatment with synbiotics and FMT demonstrated controversial results due to the limited data. In heart diseases, an in vivo study showed that rats treated with probiotics and prebiotics containing *Lactobacillus plantarum* 299 v could reduce infarct size and improve left ventricular function before coronary artery ligation.^352^ Gan et al. demonstrated similar cardioprotective results in a rat model of myocardial ischemia after supplementation with *Lactobacillus rhamnosus* GR-1.^353^ In addition, modulation of the gut microbiota through probiotics may present potential therapeutic strategies to protect against lung diseases.^354^ Moreover, probiotics is believed to have some positive effect on COVID-19 treatment. For example, Chen et al. suggested that probiotics could reduce hyperinflammation from COVID-19 through its anti-inflammatory effects.^355^ However, a systematic review by Bafeta et al., which investigated 384 RCTs, found that the report of side effects in published RCTs assessing probiotics, prebiotics, and symbiotic is often lacking or inadequate.^356^ Therefore, the safety of these interventions cannot be determined without enough safety data.

#### Antibiotic

Antibiotic administration is the most common approach to manipulate the composition of the gut microbiota. Researchers found that modulation of gut microbiota by antibiotics improves insulin signaling in high fat-fed mice.^357^ In a study of melanoma and lung cancer models, vancomycin can enhance the anti-tumor response induced by radiotherapy in mice by increasing CD8 + T cell infiltration and IFN-γ expression.^358^ Many studies have shown that antibiotics can prevent cancer development or attenuate tumor proliferation. For example, Bullman et al. showed that metronidazole treatment can eradicate the colonization of *Fusobacterium* and ameliorate the progression of CRC.^359^ The results showed that colonization of *Fusobacterium* with CRC tumor cells was maintained in distal metastasis, demonstrating a stable microbiome composition between primary and metastatic tumors. In addition, antibiotic treatment that reduced *Fusobacterium* load also inhibited cancer cell proliferation and tumor growth. Therefore, this finding suggests the potential of microbiota modulation, i.e., antibiotic intervention, for patients with microbial-associated cancer. However, we cannot exclude the possibility that broad spectrum antibiotics may have negative impact on the healthy intestinal microbiota, therefore the use of antimicrobial agent targeting to the specific bacteria is highly important. At the same time, antibiotics also showed potential as immunotherapy. In a metastatic mouse model, antibiotic consumption of the gut microbiota could inhibit tumor growth by triggering an anti-tumor immune response.^360^ The researchers studied the effect of antibiotics on tumor growth of pancreatic cancer, CRC, and melanoma. It is found that gut microbiota depletion by oral microbiota significantly reduced the tumor growth in all tumor models. Interestingly, the inhibition effect was not observed in Rag-1 KO mice, which lack mature B and T cells, suggesting the effect may be dependent on host immunity. Indeed, the mechanistic study showed that gut microbiota depletion resulted in a significant increase in IFγ producing T cells and the decrease in IL-17A/IL-10 producing T cells. In addition, gut microbiota depletion led to infiltration of effector-T cells into pancreatic tumors. Previous studies have shown that immune checkpoint inhibitors failed to antagonize pancreatic cancer due to low effector-T cell infiltration. Hence, the antibiotic treatment may be beneficial as an adjuvant therapy along with conventional immunotherapy. In patients with early gastric cancer, the eradication of *H. pylori* through the combination of amoxicillin and clarithromycin is associated with a lower incidence of metachronous gastric cancer and improvement of the degree of gastric gland atrophy.^361^

It remains controversial that, although antibiotics can effectively eradicate pathogens or harmful bacteria, their non-selective antibacterial effects may kill the symbiotic microbes, leading to another ecological disorder. It may also impair the efficacy of cancer immunotherapy and lead to treatment resistance. Vétizou et al. demonstrated that the efficacy of CTLA-4 blockade was associated with the T cell response for *B. fragilis* in mice and patients. Moreover, while GF mice were not responding to CTLA-4 blockade, introduction of *B. fragilis* was able to overcome this defect.^362^ Therefore, this study suggests a key role of microbiota in triggering the response to immunotherapy and it is meaningful to explore if other bacteria have the similar functions. Hernández et al. reported that compared with untreated individuals, subjects receiving antibiotics showed greater or unbalanced sugar anabolic capacity.^363^ Clinical studies have shown that antibiotics are closely related to the increased risk of CRC development,^364,365^ though this result may be affected by confounding indications.^366^ For example, compared with cancer patients, immunodeficient patients are more susceptible to bacterial infections that require antibiotic treatment.^364^ Despite the controversy surrounding the use of antibiotics, their potential for microbiota regulation should not be underestimated. With the development of modern sequencing methods, we can have a more comprehensive understanding of the impact of antibiotics on microbial communities, thereby bringing new vitality to the use of antibiotics.

### Fecal microbiota transplantation

FMT refers to a method of introducing a solution of fecal matter from a donor into the intestinal tract of a recipient to cure disease. FMT treatment, which was first documented in China in the 4^th^ century,^367^ will change the recipient’s microbial composition directly. The most prominent results in the use of fecal transplantation for disease treatment have been in the treatment of recurrent Clostridium difficile infection (rCDI), with reported cure rates near 90%.^368^ In 2013, FMT was approved by the FDA to treat rCDI. FMT methods include the use of a naso-intestinal tube, gastroscopy, and colonoscopy, all with different efficacies. A meta-analysis conducted by Laniro et al. showed that capsule FMT has an overall response rate of more than 90% and is minimally invasive.^369^

So far, more than 100 case reports and clinical trials of FMT for rCDI have been published; most reports have high resolution of diarrhea associated with rCDI. Several meta-analyses have confirmed that FMT is superior to standard antibiotic therapy. They also showed that FMT is a safe treatment method for patients with rCDI.^370^ Compared with the traditional therapy of vancomycin regimen which is only 31% effective, FMT therapy showed a cumulative effectiveness of 94%.^371^ The clinical remission rate of FMT therapy in the RCT study of UC is about 36–37%. FMT is also widely investigated in the treatment of cancer,^372^ diabetes,^373^ ASD,^266^ multiple sclerosis,^374^ atherosclerosis and hypertension,^375^ graft vs host disease,^376^ Parkinson’s disease,^377^ hepatic encephalopathy and NAFLD.^378^ Although these treatments showed promising results, they were investigated in preclinical models, or the sample sizes were too small. Therefore, extensive studies are required before drawing further conclusions.

Currently, there are several mechanisms proposed for FMT including the following: 1) FMT may stimulate decolonization of pathogenic microbes and enhance host resistance to pathogens by direct ecological competition;^379^ 2) repopulating gut microbiota by FMT helps to restore immune function and reduce host damage induced by abnormal microbial colonization of the gastrointestinal tract; 3) FMT facilitates the restoration of essential metabolites used for host metabolism, including SCFAs, antimicrobial peptides, bacteriocins, and bile acids.^380^ FMT is safe to a large extent, and large studies report mainly minor, short-lived adverse reactions. The specific high-risk population is mainly immunocompromised patients.^381^ But this therapy still faces many challenges. For example, regarding the standardization of donor screening, eligible stool donors are often rare if considering the risk of infection. Starting from the selection of a donor to the route of administration and dynamic monitoring after FMT treatment, the entire FMT process requires the use of personalized methods to reach its full potential. Therefore, the future development direction of FMT may be in precision medicine.^382^

### Engineering gut bacteria

Most bacteria that coexist with humans are nonpathogenic. Advances in modern DNA technology have made it possible to engineer bacteria for disease treatment. Based on traditional genetic engineering methods, engineered probiotics have been used in the treatment of colitis, diabetes, obesity, and a large number of pathogenic infections.^383–385^ *Lactobacillus jannaschii* (a conventional flora of the female vagina) has been modified to secrete HIV-resistant cyanovirin-N protein. This engineered bacteria has been proven to reduce HIV infection by 63% in rhesus monkeys.^386^ There are different types of engineered bacterial therapies for diseases, such as synthetic immune regulatory proteins, chemotactic response systems, and protein delivery systems.

“Smart probiotics” created using genetic engineering technology brings vitality to the application of probiotics. It has a better efficacy than natural probiotics. For example, *Lactococcus lactis* expressing human Trefoil Factor 1 (a cytopeptide involved in epithelial wound healing) has been formulated as a mouthwash for the treatment of oral mucositis.^387^ A combination therapy of engineered *Lactococcus lactis* has been used in a clinical trial of T1DM treatment.^388^ Moreover, an engineered *Lactococcus lactis* strain which secretes the anti-inflammatory cytokine IL-10 showed a clinical benefit in CD.^389^ Insulin production in epithelial cells can be induced by the gut hormone GLP.^390^ Duan et al. reported that an engineered GLP-1-secreting *Lactobacillus gasseri* strain can reprogram intestinal cells into insulin-secreting cells.^391^

Bacteria can bypass problems associated with poor selectivity and limited tumor penetrability of conventional cancer chemotherapies and can be finely engineered to sense and respond to the tumor microenvironment.^392^ One strategy is to utilize the native bacterial cytotoxicity to kill cancer cells. For example, *Clostridium* and *Salmonella* has exhibit anticancer effect in mice models. Accumulation of the bacteria in tumor tissues will induce neutrophil infiltration and antitumor immune response. Such response was also observed in phase 1 clinical trial that administrated a modified *Salmonella* strain to patients with metastatic melanoma.^393^ The second strategy is using engineered bacteria to directly express anticancer agents or transfer eukaryotic expression vectors into cancer cells.^394^ With these approaches, the bacteria can 1) generate cytotoxic agents such as Cytolysin A to induce cancer cell apoptosis, 2) deliver cytokines such as IL-2, TNFSF14 that activate immune cells to eradicate cancer cells, and 3) sensitizing immune system against cancer cells by expressing tumor antigen. The third strategy is using bacteria to transfer genetic material to cancer cells. Therefore, it stimulates competition with the mechanisms that foster tumor formation, through the in-situ delivery of polypeptides with pro-apoptotic activity, anti-angiogenic factors, and cytokines. Bacteria have also been engineered to silence the expression of important genes related to tumor development through RNA interference. For instance, Xiang et al. reported that *E. coli* can be engineered to transfect host cells with plasmids encoding short-hairpin RNAs (shRNAs) silencing catenin beta-1, whose overexpression is involved in several types of cancer.^395^ This therapy has been granted orphan drug status by the FDA for the treatment of familial adenomatous polyposis and is currently under clinical trial investigation to analyze the safety and tolerability.

Gene-editing technology such as CRISPR has broadened the application of engineered bacteria in microbiota modulation. CRISPR is being utilized in the development of novel antimicrobial strategies. Hwang et al. showed that the exonuclease CRISPR-associated protein 3 (Cas3) can be engineered into a probiotic, which has the capacity to efficiently kill pathogenic bacteria.^396^ A bacterial protein secretion system (T3SS) can transfer proteins into the cytoplasm of infected cells. With this system, engineered bacteria can carry polypeptide vaccines or proteins into host cells and carry transcription factors into the cell. In addition, dysregulation of the microbiome can lead to cytokine storms, which may be associated with a decrease in angiotensin 2 (ACE2).^397^ Based on this, Verma et al. developed an expression and delivery system (LP) using probiotic species *Lactobacillus piracies* as a live vector for oral delivery of human ACE2. It provides a new strategy for correcting the imbalance of the gut microbiota while increasing the serum ACE2 level.^398^ It is true that the way in which a given supplement or drug affects the microbiota-host interface is obviously not enough for the complex human environment. Awareness of the range of possible interactions between the intervention and the host’s diet, genome, immune system, and resident symbionts should be taken into consideration. Although the clinical application of new technologies, such as T3SS and CRISPR, still require more investigation, they provide more opportunities and possibilities for microbiota therapy in the future.

### Gut microbiota and drug response

It is well established that drug response, mainly characterized by pharmacokinetic (PK) and pharmacodynamic (PD) properties, may differ among individuals due to factors such as gender, age, and genetic variations.^399^ However, it was only recently that researchers identified microbiota as a mediator of drug response, highlighting its role in medical therapy. We herein describe the role of gut microbiota on modulating drug effect as it is a current research focus.

It is known that the gut microbiota can metabolize a wide range of substances, which can have potential implications for affecting drug absorption. Particularly, the stability of orally administered drugs can be affected in the GI tract before entering the systemic circulation. Sousa et al. summarized that over thirty drugs are substrates of bacterial enzymes in the distal gut.^400^ Recently, it was suggested that small intestine microbiota may also have profound impact on host physiological functions.^401^ This finding highlighted the potential drug-microbiota interaction, since small intestine is a major site for drug absorption. Indeed, numerous studies have reported altered drug PK mediated by gut microbiota with clinical implications. For example, Sun et al. reported that a hypoxic environment can affect the composition of gut microbiota, which led to increased absorption of aspirin in rats.^402^ Matuskova et al. identified that concomitant orally administrated probiotic *E. coli* strain Nissle 1917 (EcN) affected the PK of the antiarrhythmic drug amiodarone in male rats.^403^ EcN increased the plasma level of amiodarone metabolites, probably due to increasing the drug absorption or the activity of CYP2C enzymes, which was not observed in the reference non-probiotic strain.

Wallace et al. suggested that β-glucuronidases from E. coli can metabolize irinotecan into the active metabolite SN-38 in intestinal lumen and damage the intestinal epithelium in a mouse model.^404^ Lindenbaum et al. reported that the most widely used cardiac glycoside digoxin can be converted to reduced derivatives produced by Eubacterium lentum, a common gut anaerobe.^405^ The same research group reported a follow-up study that showed that administration of antibiotics erythromycin or tetracycline was able to significantly reduce the levels of digoxin, reduced metabolites, and increase serum digoxin level to a maximum of 2-fold. Wu et al. established a pseudo-GF diabetic rat model to investigate the relationship between metformin and gut microbiota.^406^ They found that the antihyperglycemic effect of metformin was reduced by more than 40% in gut microbiota-depleted group. Moreover, the hepatoprotective effect of metformin was significantly reduced in the absence of gut microbiota. Recent studies in IBD revealed that gut microbiota may influence the metabolism of IBD drugs mesalazine, methotrexate, thioguanine, and glucocorticoids.^407^ In particular, thioguanine can be converted to its active form by gut bacteria without the requirement of host metabolism. Studies also suggested that microbiota can affect host metabolism by modulating cytochrome P450 enzymes and UDP-glucuronosyltransferase.^408^ Interestingly, a recent study by Klünemann et al. established multiple new bacteria-drug interactions, with more than half of them ascribed to bioaccumulation,^409^ phenomenon by which bacteria can store the drug without chemical modification, thereby altering host drug response. Particularly, the behavioral response of *Caenorhabditis elegans* to antidepressant duloxetine was attenuated by bioaccumulating bacteria such as S*. salivarius*.

A large number of data confirmed that the gut microbiota can have a major impact on drug PK and subsequently the drug response in clinical settings. A better understanding of such interaction is required to develop effective treatment strategies.

## Conclusion

After decades of research, we have gradually established a new role of microbiota in health and disease. It is now confirmed that microbiota can affect almost all aspects of the host, while its dysbiosis is related to a wide spectrum of diseases. Thanks to advanced research technologies, we are able to closely examinate how microbiota maintain human health and contribute to pathogenesis. However, the study of microbiota is mainly focused on the bacterial component; the role of fungi, viruses, and other microbes in health and disease remain largely inconclusive. In addition, while microbiota dysbiosis is often observed in disease states, the causative role of microbiota is yet to be established. Hence, there are still a lot of questions to be answered in this field. The greater understanding of this host-microbiota relationship has allowed for the development of microbiota-based therapy such as FMT and bacteria modulation. These strategies are well on the way to achieving the optimal clinical effect in the treatment of C. difficile infection, diabetes, inflammatory bowel disease, etc. In summary, we are now in a better position to treat diseases and foster health via manipulation of the microbial symbionts.

## Supplementary information

Supplementary Table 1
