# Supplementary material for: Microbiota in health and diseases
Source: Signal Transduct Target Ther. 2022 Apr 23;7:135. doi: 10.1038/s41392-022-00974-4 (PMC9034083; doi:10.1038/s41392-022-00974-4)
Supplement: Supplementary file 1 — Supplementary Table 1 [file 41392_2022_974_MOESM1_ESM.pdf]

Table S1. Summary of microbiota-related clinical trials

Disease	NCT#	Total patient (n)	Treatment group (n)	Response	No response
Crohn's disease	NCT02097797	17	9	4	5
	DOI:10.1007/s10620-019-05751-y	27	27	18	9
	NCT01757964	9	9	7	2
	NCT01793831	139	174	131	43
	NCT01793831	25	25	17	8
Ulcerative Colitis	ACTRN12613000236796	73	73	15	58
	NCT01545908.	75	38	9	29
	NCT01650038	50	50	37	13
	CTRI/2018/02/012148	31	31	27	4
	ChiCTR2000030080	20	10	9	1
	DOI: 10.1038/ismej.2017.44	34	34	12	22

	NCT01560819	10	9	7	2
	NCT02390726	12	6	3	3
	NCT02575040	30	30	21	9
	ChiCTR-ONH-17012572	16	16	14	2
C. difficile infection	NCT02743234	64	24	22	2
	NCT01703494	46	46	35	11
	DOI: 10.1007/s10620-017-4833-2	34	11	10	1
	NCT01704937	20	20	14	6
	DOI: 10.1007/s15006-016-8305-y	16	16	11	5
Cancer	DOI: 10.1007/s00520-018-4216-z	2982	390	278	112
	NCT04040712	20	10	10	0
	DOI: 10.1016/j.nut.2018.06.004	73	39	27	12
